# Protein Quality Control Systems and ER Stress as Key Players in SARS-CoV-2-Induced Neurodegeneration

**DOI:** 10.3390/cells13020123

**Published:** 2024-01-09

**Authors:** Elena Gavilán, Rafael Medina-Guzman, Bazhena Bahatyrevich-Kharitonik, Diego Ruano

**Affiliations:** 1 Departamento de Bioquímica y Biología Molecular, Facultad de Farmacia, Universidad de Sevilla (US), 41012 Sevilla, Spain; rafmedguz@gmail.com (R.M.-G.); bahatyrevich@gmail.com (B.B.-K.); ruano@us.es (D.R.); 2 Instituto de Biomedicina de Sevilla, IBIS, Hospital Universitario Virgen del Rocío, Junta de Andalucía, CSIC, University of Seville (US), 41013 Sevilla, Spain

**Keywords:** COVID-19, SARS-CoV-2, protein quality control systems, ER stress, neurodegeneration

## Abstract

The COVID-19 pandemic has brought to the forefront the intricate relationship between SARS-CoV-2 and its impact on neurological complications, including potential links to neurodegenerative processes, characterized by a dysfunction of the protein quality control systems and ER stress. This review article explores the role of protein quality control systems, such as the Unfolded Protein Response (UPR), the Endoplasmic Reticulum-Associated Degradation (ERAD), the Ubiquitin–Proteasome System (UPS), autophagy and the molecular chaperones, in SARS-CoV-2 infection. Our hypothesis suggests that SARS-CoV-2 produces ER stress and exploits the protein quality control systems, leading to a disruption in proteostasis that cannot be solved by the host cell. This disruption culminates in cell death and may represent a link between SARS-CoV-2 and neurodegeneration.

## 1. Introduction

The Coronavirus Disease 2019 (COVID-19) pandemic, produced by the Severe Acute Respiratory Syndrome Coronavirus 2 (SARS-CoV-2), had an irrevocable impact on the worldwide environment, causing significant disruptions to both livelihoods and economies.

SARS-CoV-2’s structure and mechanism of infection have been well characterized [1,2,3,4,5]. The virus comprises a lipid envelope studded with spike (S) proteins. These spikes facilitate viral entry into host cells by binding to angiotensin-converting enzyme 2 (ACE2) receptors on the cell surface. Following attachment, the virus enters the cell by endocytosis. Its genetic material consists of a single-stranded RNA molecule, which encodes structural proteins, non-structural proteins (NSP), and accessory proteins. Once inside, the viral RNA is translated into proteins, including those for replication and the formation of new virus particles [1,2,3,4,5].

While initially characterized by respiratory symptoms, it has become increasingly evident that SARS-CoV-2 possesses a multifaceted nature, affecting multiple organs and tissues, including the nervous system, even in people that experienced mild or asymptomatic infection [6,7,8]. Therefore, aside from the already successful development of vaccines, it is extremely necessary to understand the cellular and molecular mechanisms that orchestrate these long-term consequences and to find therapies to reduce and/or avoid their appearance and progression. Reports of neurological complications associated with COVID-19 have emerged from across the globe, raising questions about the virus’ potential impact on the central nervous system (CNS) [9]. These neurological complications include a wide spectrum, from mild symptoms like anosmia and ageusia to more severe manifestations, including encephalopathy, seizures, and stroke [6]. Moreover, accumulating evidence has suggested a potential link between SARS-CoV-2 infection and the exacerbation or even initiation of neurodegenerative processes, such as Parkinson’s and Alzheimer’s diseases (AD and PD, respectively) [10,11,12], underscoring the critical need to unravel the fundamental mechanisms involved. The mechanisms underlying these neurological manifestations are multifaceted and may involve both direct viral invasion and indirect immune-mediated responses. Solving this intricate puzzle is imperative not only for comprehending the full spectrum of COVID-19, but also for devising targeted strategies to mitigate its neurological consequences. Of particular concern are the alterations made to the protein quality control systems after they are hijacked by the virus. These alterations and the generation of ER stress are hallmarks of both neurodegenerative pathologies (characterized by protein accumulation and aggregation) and viral infections, including SARS-CoV-2 infection. This review undertakes a comprehensive exploration of the intersection between SARS-CoV-2 and protein quality control systems. We seek to offer a broad overview of this relationship and shed light on the possible association with neurodegeneration. Specifically, we explore the current state of knowledge regarding:

  i) The critical role of protein quality control systems, such as the Unfolded Protein Response (UPR) induced by Endoplasmic Reticulum (ER) stress, the Endoplasmic Reticulum-Associated Degradation (ERAD) and the Ubiquitin-Proteasome System (UPS), the autophagic–lysosomal pathway and the molecular chaperones;
 ii) How these systems are manipulated during SARS-CoV-2 infection and potential therapeutic strategies targeting the viral manipulation of the protein quality control systems;
iii) How ER stress and the manipulation of the protein quality control systems induced by SARS-CoV-2 in the central nervous system (CNS) could lead to neurodegeneration.

## 2. Role of Protein Quality Control Systems in SARS-CoV-2 Infection

Protein quality control systems are essential for keeping the balance in how proteins are made, folded, and removed in cells [13]. Viral infections, such as SARS-CoV-2, can disrupt these systems and induce ER stress, affecting cellular functions. This discussion provides an overview of key protein quality control mechanisms, including the UPR, ERAD and UPS, as well as autophagy and molecular chaperones. All these systems are not independent, but are interconnected, so an effect on one of them can have consequences for the others [13]. We explore how viruses interact with these systems, focusing on SARS-CoV-2. Additionally, we examine how dysfunctions in protein quality control are common in both neurodegeneration and viral infections, suggesting a potential link. Understanding these connections is crucial for deciphering how viral infections, especially SARS-CoV-2, contribute to neurodegeneration, and for developing targeted therapies to address related proteostasis issues.

### 2.1. ER Stress and UPR

ER stress and UPR are common aspects that emerge during the appearance and progression of both neurodegenerative diseases [14,15,16] and viral infections [13,17]. Viral infections often trigger an upsurge in protein synthesis, potentially surpassing the folding capacity of the ER. Consequently, this imbalance leads to the accumulation of unfolded proteins, inducing ER stress [13,17]. In response to ER stress, cells activate a multifaceted signaling network known as UPR, an adaptive response aimed at mitigating the burden of unfolded proteins to sustain cellular viability and function [18,19].

**Figure 1:**
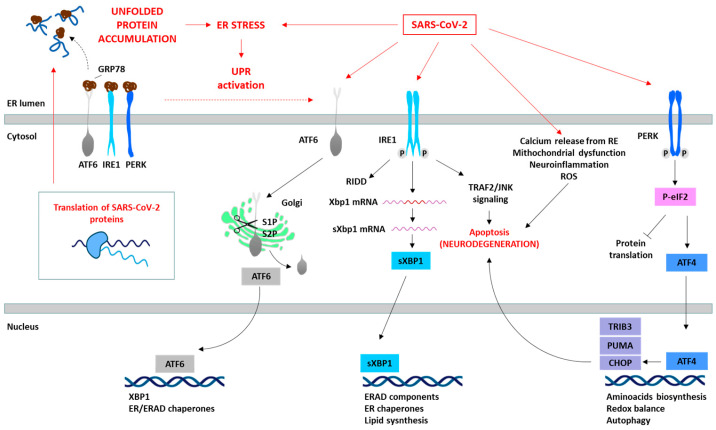
**SARS-CoV-2 induces ER stress and activates the UPR.** The translation of SARS-CoV-2 proteins generates ER stress, which activates the UPR as an antiviral host cell response. However, SARS-CoV-2 is able to manipulate the different pathways of the UPR (red arrows) depending on the viral requirements for its replication. The specific proteins involved in the SARS-CoV-2 and UPR interaction are summarized in Table 1. The prolonged activation of the UPR leads to the initiation of pro-apoptotic signaling pathways (e.g., IRE1/TRAF2/JNK or PERK/ATF4/CHOP), disturbances in cellular calcium levels due to ER release, mitochondrial dysfunction, and consequently ROS generation, and the induction of oxidative stress, culminating in cell death.

The UPR is a sophisticated signaling pathway initiated by the activation of three primary UPR stress sensors (see Figure 1): inositol-requiring protein 1 (IRE1), protein kinase RNA-like ER kinase (PERK), and activating transcription factor 6 (ATF6) [13,18,20,21,22]. The glucose regulated protein-78 (GRP78, also known as BiP or HSPA5), is an ER chaperone that plays a pivotal role as the principal regulatory protein by binding to these sensors. In the absence of stress, GRP78 predominantly associates with these three proteins, effectively suppressing their activity and preventing the initiation of UPR signaling. However, under stress conditions characterized by the accumulation of misfolded proteins in the ER (as observed in neurodegenerative diseases and in viral infections), GRP78 interacts with these unfolded proteins, aiming to maintain their foldable state and resulting in the release of the three UPR mediators (Figure 1). The specific mechanisms of the UPR pathways include [20,21]:

− IRE1 activation and splicing of XBP1 (X-box binding protein 1) mRNA, resulting in the production of sXBP1, an active transcription factor. sXBP1 regulates the expression of chaperones and ERAD components, reinforcing ER’s protein-folding and -degradation capacity. IRE1 also catalyzes the degradation of a large number of mRNAs and some pre-microRNAs (pre-miRNAs). This process is called regulated IRE1-dependent decay (RIDD) [23,24]. On the other hand, it is known that IRE1 is capable of forming high-order complexes in the ER membrane and interacting with a large number of proteins, among which the tumor necrosis factor (TNF) receptor-associated factor 2 (TRAF2) stands out. This interaction activates a cascade of signaling that leads to the activation of c-Jun N-terminal kinase (JNK), which, in turn, can inhibit some anti-apoptotic members of the BCL-2 family while activating pro-apoptotic proteins. Together, these two events lead to the oligomerization of BCL-2-like protein 4 (BAX) and BCL-2 antagonist/killer (BAK), initiating the apoptosis process [23,24,25];
− PERK activation and the phosphorylation of the eukaryotic initiation factor 2α (eIF2α), a pivotal regulator of protein translation. Phosphorylated eIF2α reduces global protein synthesis, thereby alleviating the ER burden and allowing cells to cope with ER stress. However, this process can also induce apoptosis by upregulating the C/EBP homologous protein (CHOP). The prolonged upregulation of CHOP induces apoptosis through pathways involving the BCL2 binding component 3 (BBC3/PUMA) and the tribbles pseudokinase 3 (TRIB3).
− ATF6 activation and translocation to the Golgi apparatus, where it is cleaved by site-1 (S1P) and site-2 (S2P) proteases, releasing a 50 kDa N-terminal fragment that translocates to the nucleus. This fragment acts as a transcription factor that subsequently upregulates genes encoding ER chaperones and other proteins involved in ER quality control.

These sensors orchestrate adaptive processes through both transcriptional and non-transcriptional responses to restore ER homeostasis (Figure 1). This involves adapting protein synthesis, enhancing protein folding capacity, and increasing the efficiency of ERAD [20,21,22]. The activation of these three UPR pathways leads to diverse downstream consequences, depending on the nature and intensity of the stimuli and the specific cell type involved. Although the primary objective of the UPR is to reinstate ER homeostasis, prolonged or severe ER stress can activate apoptotic pathways through factors such as activating transcription factor 4 (ATF4) and CHOP, ultimately culminating in cell death, as observed in neurodegenerative processes [13,20,21,22].

#### 2.1.1. ER Stress and UPR in Viral Infections

**Figure 2:**
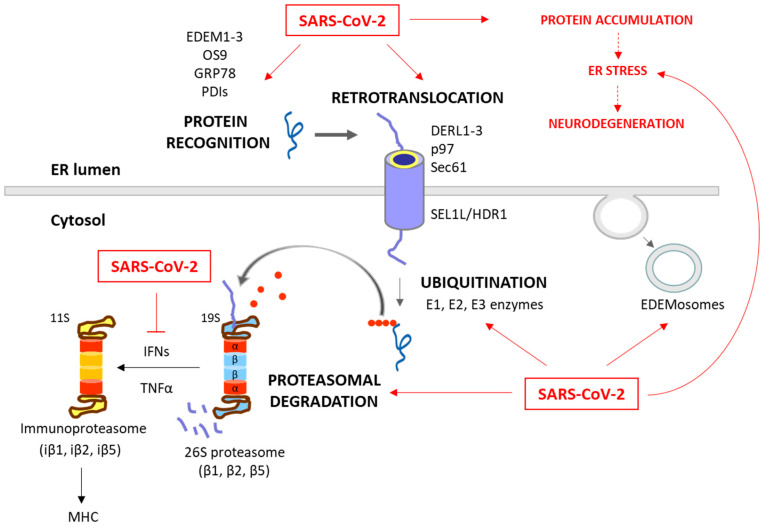
**Degradation of unfolded protein through the ERAD/UPS pathways and SARS-CoV-2 interplay.** When chaperones are not able to achieve the proper folding of proteins within the ER, these misfolded proteins are recognized and isolated by lectins and ERAD components, including EDEM1-3, OS9, PDIs and GRP78. The targeted proteins are then recruited to the ERAD complex and retrotranslocated to the cytosol. This complex involves Sec61, DERL1-3 and p97, together with SEL1L and HRD1, which assist in the ubiquitination process. After (poly)ubiquitination, the proteins are recognized by the 26S proteasome and deubiquitinated for degradation. While these processes are common antiviral responses of host cells, SARS-CoV-2 is able to positively or negatively manipulate this machinery to ensure its replication, escape and survival (red arrows). Consequently, the ERAD and UPS systems are not efficient in solving protein accumulation, leading to neurodegeneration. Moreover, SARS-CoV-2 can inhibit IFN production, and therefore the formation of immunoproteasome and antigen presentation. The proteins responsible for the ERAD/UPS and SARS-CoV-2 interplay are indicated in Table 2.

**Figure 3:**
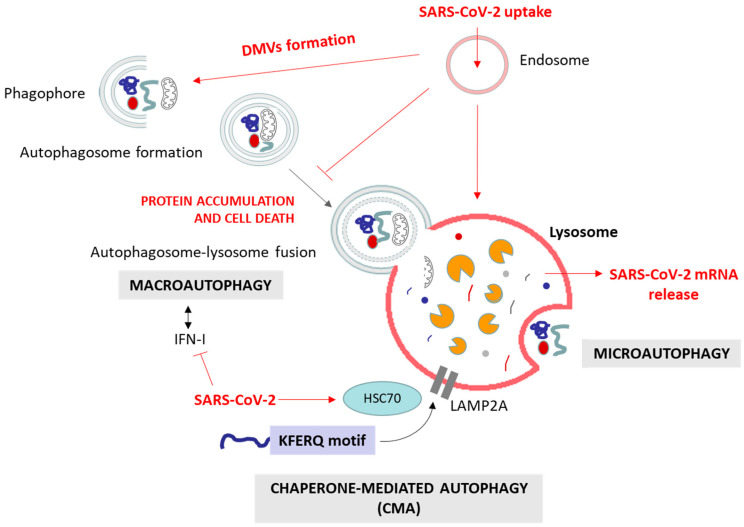
**Main types of autophagy (macroautophagy, microautophagy and CMA) and SARS-CoV-2 points of interaction.** The virus reaches the lysosome through the endosomal pathway, and its mRNA is released to the cytosol. During its life cycle, SARS-CoV-2 can induce or block autophagy and autophagosome formation, suppress autophagosome–lysosome fusion, and use the endosomal and autophagosomal machinery to form DMVs, facilitate its replication, evade immune responses, and promote its survival within host cells. Similarly, the virus can block the IFN-I-dependent induction of autophagy. The proteins involved in this interplay are detailed in Table 3. The inhibition of autophagosome–lysosome fusion yields the accumulation of proteins and substrates targeted for autophagy degradation.

It has been extensively described that viruses use the ER for entering, replicating and assembling, but also to evade the host immune system [26,27]. Viral infections produce ER stress (especially those viruses that interact with the ER for their replication). When the hijacked host cell machinery synthesizes viral proteins at a rapid rate, it can overwhelm the ER’s capacity to fold these proteins correctly. This excessive load of misfolded proteins leads to ER stress, which subsequently initiates compensatory pathways. Actually, a total of 35 animal viruses (mainly RNA viruses) have been documented to induce ER stress [28]. The progression of ER stress and the activation of the UPR (as well as the ERAD/UPS systems) can be involved in both the host defense mechanism and viral pathogenesis [29]. On the one hand, viral proteins induce ER stress, which activates the UPR (Figure 1), as part of the host cell defense response [30]. This includes the activation of ER chaperones and the stimulation of all three UPR sensors (ATF6, IRE1 and PERK), as well as the ERAD pathway (Figure 2), enhancing the degradation of viral particles to restore the homeostasis [31,32]. Also, the viral induction of ER stress and the activation of the UPR can induce the inflammatory response and activate other protein quality control systems, such as autophagy (Figure 3) [17]. On the other hand, to ensure their survival and pathogenesis, viruses have developed intricate mechanisms to modulate signaling pathways related to ER stress, UPR and immune responses for their own benefit [28]. Some examples are the Hepatitis C virus (HCV), which activates the eIF2α/ATF4 pathway, leading to the selective degradation of HCV pro-oxidant proteins Core and NS5A [33]; the Zika virus (ZIKV) activates IRE1, promoting XBP1 mRNA splicing and increasing viral replication [34]. However, the Herpes simplex virus (HSV-1) suppresses the UPR initially (especially the IRE1 pathway and XBP1 activation), and enhances it later during infection, aligning with the replication stages [19,35]. Also, the Porcine delta coronavirus (PDCoV) is able to improve viral replication through the ATF6 pathway, while PERK signaling abrogates it, and IRE1 is not involved in this viral replication [36]. Hence, the manipulation of distinct UPR branches, and subsequently the potential therapeutic approaches, varies according to the specific virus.

#### 2.1.2. ER Stress and UPR in SARS-CoV-2 Infection

##### ER Stress/UPR-Related Antiviral Strategies against SARS-CoV-2

After entering the cell by binding its spike protein to ACE2 receptors, essential stages of the virus’ life cycle take place within the ER. These encompass critical processes such as protein synthesis, modification, genome replication, and assembly [19]. Accordingly, infection with SARS-CoV-2 induces ER stress and consequently activates the UPR, ERAD and autophagy (Figure 1, Figure 2 and Figure 3) in different tissues, including the brain [37,38,39]. The activation of these protein quality control systems is part of the antiviral response of host cells to SARS-CoV-2 infections (Figure 1). However, even if the UPR is activated to deal with the harmful effects of the virus, this activation can induce either cell survival or apoptosis, depending on the severity and duration of the stress [37,40]. Liu et al. (2022) observed in whole-blood samples (containing multiple cell types) of COVID-19 patients that altered miRNAs, as an antiviral host response against SARS-CoV-2, were primarily linked to ER stress and UPR pathways’ regulation (including ATF6, IRE1 and PERK pathways), as well as to the generation of proinflammatory cytokines and immune responses [40].

##### ER Stress/UPR-Related SARS-CoV-2 Strategies

As expected, the virus can manipulate these systems for its own replication [39,41,42,43,44,45] by interacting with different ER stress/UPR components, as summarized in Table 1. The origin of these alterations, whether initiated by the host cell or the virus, remains unclear, posing a challenge in understanding this complex interaction (Figure 1). Researchers continue to explore these mechanisms in attempts to develop targeted therapies against COVID-19.

The manipulation of these processes by SARS-CoV-2 seems to depend on the type of cell or tissue. Bartolini et al. (2022) demonstrated in SARS-CoV-2-infected VERO-E6 cells that ER stress and UPR signaling (mainly via the IRE1/TRAF2 pathway) potentially contribute to viral replication and lead to inflammatory processes related to viral pathogenesis [41]. Treatment with Nelfinavir reduced IRE1α activity and restored host cell balance, highlighting UPR signaling and ER stress as vital in SARS-CoV-2 interactions and inflammation. Other authors, such as Chaudhry et al. (2022), showed that ER stress and the activation of the PERK pathway of the UPR played key roles in SARS-CoV-2 infection in dopamine-containing neurons (dDCNs) derived from a human neural progenitor cell line (ReNVM) [42]. Besides this, elevated ER stress (indicated by higher GRP78 levels), increased phosphorylated eIF2α (PERK activation) and cell death upon overexpressing the SARS-CoV-2 spike protein in HEK293T cells (derived from the human embryonic kidney 293 cell line) have been observed [19]. Moreover, within these cells, the SARS-CoV-2 open reading frame 8 (ORF8) protein, distinctive to this virus, initiated ER stress and UPR activation. Specifically, it predominantly activated the ATF6 and IRE1 branches. This activation led to the upregulation of proteins such as GRP78 and the protein disulfide-isomerase A4 (PDIA4), alongside other stress-related effectors such as CHOP, ER Degradation-Enhancing α-Mannosidase-Like Protein (EDEM)**,** and Derlin-3 (DERL3). Ultimately, this orchestrated response facilitated the replication of SARS-CoV-2 [39,43,44]. Furthermore, Wen-Qing et al. (2021) found that SARS-CoV-2 protein ORF3a activates the three UPR pathways in human cervical cancer-derived HeLa cells, increasing key protein levels related to viral replication [45]. UPR modulators of ATF6, IRE1 and PERK pathways have been evaluated as potential therapeutic targets against COVID-19, reducing the viral load and alleviating the associated pathophysiology [43,46]. Examples such as PERK inhibitors, GSK2606414, ISRIB and Salubrinal reduced viral replication [43,47]. The inhibition of IRE1 by compounds such as STF-083010, and ATF6 by inhibitors like AEBSF, demonstrated promising efficacy against SARS-CoV-2. These inhibitors have shown the potential to significantly reduce the virus titer, suggesting a potential avenue for therapeutic intervention [43]. Additionally, thapsigargin, an ER stress inducer, effectively inhibits the replication of coronaviruses, including Human coronavirus 229E (HCoV-229E), Middle East respiratory syndrome coronavirus (MERS-CoV), and SARS-CoV-2 [48]. It reverses virus-induced translational shut-down, improves cell viability, and counteracts the CoV-mediated downregulation of IRE1 and GRP78.

**Table 1:** Summary of reported ER stress/UPR components hijacked by SARS-CoV-2.

	Key Components	SARS-CoV-2 Strategies	References
**ER Stress and UPR**	ATF6	Activated by SARS-CoV-2 ORF8 protein.	[36,40,41]
	IRE1α	Contributes to viral replication. Activation in dDCNs. Activated by SARS-CoV-2 ORF8 protein.	[36,38,39,40,41]
	PERK	Activation plays a key role in SARS-CoV-2 infection in dDCNs. Reduced viral replication upon pharmacological inhibition.	[39,40,44]
	GRP78 PDIA4 CHOP	Upregulated by SARS-CoV-2 ORF8 protein.	[36,40,41]

### 2.2. ERAD and UPS

ERAD is a crucial process in the quality control of ER proteins, facilitating the elimination of aberrant proteins by the UPS [49]. This process is essential for maintaining cellular proteostasis, as misfolded or unassembled proteins can compromise the cell function. This process consists of three phases: recognition of misfolded or damaged proteins in the ER, retrotranslocation into the cytosol, and ubiquitin-dependent degradation by the proteasome (Figure 2). ER luminal chaperones both prevent improper folding and identify terminally misfolded proteins, directing them for ERAD. Some ERAD components include the EDEM family members, such as EDEM1, EDEM2, and EDEM3, as well as the Osteosarcoma 9 (OS9) protein, the chaperone GRP78, and protein disulfide-isomerases (PDIs), such as the ER DnaJ domain-containing protein 5 (ERdj5). These components facilitate the recognition, recruitment and disposal of the misfolded proteins [50,51] for retrotranslocation via the ERAD complex. This complex involves the Sec61 translocon, DERL1-3 and valosin-containing protein (p97), together with the suppressor enhancer Lin12 1-like (SEL1L) and the ERAD-associated E3 ubiquitin–protein ligase (HRD1) that contribute to the ubiquitination process [51,52]. After (poly)ubiquitination, the proteins are recognized and degraded by the proteasome (Figure 1).

The ubiquitination process (which can take place in the ER or in the cytosol) involves the covalent attachment of ubiquitin molecules to lysine (K) residues on the substrate protein, through an enzymatic cascade involving E1 (ubiquitin-activating), E2 (ubiquitin-conjugating), and E3 (ubiquitin ligase) enzymes. The specificity of ubiquitination is determined by the type of linkage formed between ubiquitin molecules [53]. Hence, the E3 ubiquitin ligases play a pivotal role in specifying the target proteins for degradation. Their activity, expression, and turnover are rigorously regulated to prevent inappropriate ubiquitination and maintain cellular function [49]. The type of ubiquitination will direct the protein to a specific destination. For example, K48 linkages are tagged to UPS degradation, while K63 is tagged to autophagy and non-proteolytic signaling pathways [53,54].

The proteasome, a large multi-catalytic protease, is responsible for the degradation of ERAD substrates, as well as other UPS-tagged proteins [55]. The proteasome consists of a catalytic core complex (20S proteasome) and regulatory subunits such as 19S or 11S particles. The catalytic core is the 20S proteasome, a hollow barrel-shaped structure comprising four rings with α and β subunits. The α-rings control substrate access, and the β-rings house catalytic subunits (β1, β2, and β5). This 20S proteasome degrades non-ubiquitinated misfolded or damaged proteins [13]. Additionally, the proteasome can associate with regulatory subunits, forming different types of proteasomes (mainly 26S or 30S, immunoproteasome, hybrid proteasomes) with distinct functions. The regulatory particle 19S forms the 26S or 30S proteasome when associated with the 20S proteasome. The 19S particle facilitates binding, deubiquitination, unfolding, and the channeling of target proteins for degradation [13,55]. Additionally, deubiquitinating enzymes (DUBs) can modulate ubiquitination and the ERAD process by removing ubiquitin chains from substrate proteins [56]. Besides this, cytokines such as interferon α (IFNα), IFNγ and TNFα induce the replacement of constitutive catalytic subunits (β1, β2, and β5) in the 20S proteasome with inducible subunits β1i, β2i, and β5i, and this results in the formation of the immunoproteasome (Figure 2). The immunoproteasome, expressed in immune cells, exhibits distinct proteolytic activities compared to the standard 20S proteasome. It plays essential roles in antigen presentation, γ-interferon-mediated microglial activation, cytokine production by microglial cells, and the regulation of T-cell populations, highlighting its significance in immune responses and cellular regulation [57].

The ERAD is also modulated by the ERAD tuning process, the regulation of ERAD activity by segregating ERAD components (like EDEM proteins) into specific ER-derived vesicles, named EDEMosomes (Figure 2). This segregation prevents the premature degradation of certain proteins, allowing the cell to regulate ERAD more precisely [58,59]. A huge number of associations between the ERAD pathway and human diseases have been established, highlighting the former’s significance in health and disease. Neurodegenerative diseases are characterized by disruptions in both ERAD and the UPS. These disruptions contribute to the accumulation of abnormal proteins and the formation of toxic protein aggregates, which are commonly associated with the pathogenesis of these conditions [60,61,62]. Also, beyond their fundamental roles in cell proteostasis, the ERAD and UPS have emerged as critical players in the intricate interaction between viruses and host cells during the process of viral infection [63,64,65,66,67,68,69,70,71,72,73,74,75,76,77,78,79,80,81].

#### 2.2.1. ERAD and UPS in Viral Infections

These systems also play a dual role in viral pathogenesis [82,83]. On the one hand, the ERAD/UPS mechanisms act as vital components of the host antiviral defenses by degrading viral components and activating the immune antiviral response [81,84,85,86,87,88,89,90,91]. On the other hand, many viruses employ intricate strategies to utilize the host ERAD and UPS to their advantage. They optimize their viral protein levels, facilitating viral genome uncoating, viral replication, and immune evasion. Simultaneously, the virus can target host proteins that impede viral growth [27,28,59,73,82,92,93,94,95,96,97,98,99,100,101,102,103]. This dynamic interplay between viruses and the ERAD/UPS systems highlights the fight between host defense mechanisms and viral strategies for survival and propagation.

Among the host defense events, we highlight the following ERAD/UPS-related mechanisms:

− Degradation of viral proteins. The viral proteins present in the ER lumen or the cytosol are marked for destruction, limiting viral replication within host cells [84,85,86];
− Proteasome activation. Modulating proteasome activity promotes the clearance of viral components within infected cells. Both 26S proteasome and immunoproteasome functions appear to be important for a variety of host responses to viral infection, degrading viral proteins and promoting the antigen presentation of viral particles [81,87];
− Immune response regulation. The UPS regulates immune proteins, influencing the intensity and duration of the immune response to viruses [88,89];
− Interferon response. The production of interferons under viral infection induces the expression of ERAD/UPS-related genes, enhancing the degradation of viral proteins [90,91].

In contrast, the virus life cycle depends on the host’s ERAD/UPS systems, which control protein degradation and stability during cell entry, replication, protein expression, assembly, egress, and immune evasion. Viruses can strategically target host and viral proteins for degradation by ERAD and modulate the expression or activity of ERAD-related proteins [27,28,59,92,93,94,95]. They also have the capacity to alter the selectivity of cellular E3 ligases or DUBs, which regulate an ample variety of cellular and viral targets. Alternatively, they can incorporate viral ubiquitin-like modifiers or enzymes involved in ubiquitination and deubiquitination to modify an entirely different array of substrates. The viral ERAD/UPS-related strategies can be summarized as follows:

− Degradation of host proteins. Some viruses encode specific proteins that target host proteins for degradation. For example, in the case of Human cytomegalovirus (HCMV), the viral proteins US2 and US11 induce the degradation of major histocompatibility complex (MHC) class I molecules through ERAD [94]. By eliminating MHC class I molecules, which are crucial for immune recognition, infected cells can avoid detection and destruction by the immune system. Besides, the Human Immunodeficiency Virus (HIV), through the viral protein U (Vpu), targets the host’s cluster of differentiation 4 (CD4) protein for degradation [93]. By using the ERAD pathway, Vpu facilitates the degradation of CD4, the receptor for HIV entry, thereby reducing the number of available receptors on the cell surface. This downregulation of CD4 is beneficial for the virus, as it prevents superinfection (multiple viruses infecting the same cell);
− Modulation of the expression or activity of ERAD/UPS-related proteins. Viruses can modulate the expression or activity of ERAD-related proteins to favor their replication and assembly processes. For instance, the ERAD pathway can reduce the amount of virus envelope proteins in order to control the level of virus particles, and thus facilitates chronic infections, as observed in Hepatitis B Virus (HBV) [28,95]. Zhou et al. (2022) recently published a comprehensive review on the topic of how viruses utilize the ERAD pathway to regulate their replication and propagation [92];
− Manipulation of ER membrane dynamics. Viruses can also induce alterations in ER membrane dynamics, creating a specialized membrane structures that support viral replication. This is the case of the Japanese encephalitis virus (JEV), which modifies ERAD by confiscating EDEMosomes. These vesicles segregate ERAD factors such as EDEM1, OS9, and SEL1L from the ER lumen, which fall under the control of the virus [59];
− Manipulation of ubiquitination. This viral strategy includes several possibilities—
  1. Ubiquitination of viral proteins as tools for assembly and entry. This post-translational modification serves as a molecular tag, facilitating the assembly and budding of new viral particles [82,96]. This ubiquitin-mediated process is crucial for the completion of the viral life cycle. Notably, this modification can also enhance virus–host interactions, promoting virus entry, replication, and pathogenesis [97,98,99];
  2. Manipulation of host ubiquitinating enzymes (including E1, E2 and E3 enzymes). The virus can exploit the ubiquitination of both host and viral proteins to enhance or complete their life cycles [73,100,101];
  3. Viral DUBs enzymes. As host antiviral responses are significantly dependent on the UPS function, viruses have also developed DUBs active proteins, which can facilitate viral replication and regulate the host’s innate immune response [102,103].

#### 2.2.2. SARS-CoV-2 and ERAD/UPS Interaction

In SARS-CoV-2 infections, the ERAD/UPS systems can also function as components of the host cell defense mechanisms and as an integral part of the virus’ infection strategy (Figure 2). Raaben et al. (2010) already highlighted that the UPS regulates different steps of the coronavirus infection cycle, and that ubiquitination plays a pivotal role in these processes [104]. 

##### ERAD/UPS-Related Antiviral Strategies against SARS-CoV-2

The ubiquitination level in COVID-19 patients has been proposed as a potential prognostic indicator, as well as a promising target for therapeutic intervention [105]. In this study, Che et al. (2022) utilized COVID-19 transcriptome data and bioinformatics to explore ubiquitination in COVID-19 patients. They discovered a strong association between high ubiquitination levels and favorable prognosis, as well as reduced inflammation. The SARS-CoV-2 protein ORF9b, which inhibits type I interferon (IFN-I) production and consequently host immune response, is ubiquitinated and targeted for degradation through the proteasome as a host defense mechanism [79]. Furthermore, different ubiquitin ligases have been identified as potent restrictors of SARS-CoV-2. Li et al. (2023) showed that the E3 ligase RNF5 promotes the ubiquitination and degradation of the viral envelope protein, inhibiting SARS-CoV-2 replication [69,106]. Furthermore, tripartite motif (TRIM) proteins such as TRIM25 and TRIM56, known for their involvement in maintaining cellular proteostasis and preventing protein aggregation in neurodegenerative disorders, also contribute to the defense against COVID-19 through their ubiquitin ligase activity [107,108]. The proteomic analysis of Vanderboom et al. (2021) revealed, in nasopharyngeal swab samples of COVID-19 patients, a reduction in proteasomal activity, affecting specific proteasomal subunits such as the non-ATPase subunits of the 19S regulator lid (PSMC1), proteasome 26S subunit non-ATPase 2 (PSMD2) and 7 (PSMD7), and the proteasomal ubiquitin receptor ADRM1. Concurrently, they identified an upregulation of E3 ligases and the interferon-stimulated gene 15 (ISG15) protein, a ubiquitin-like modifier, which plays a pivotal role in mediating antiviral activity by targeting viral proteins during viral infections. Moreover, the E3 ubiquitin ligases responsible for ISG15 conjugation, HERC5 and UBE2L6, were also found to be upregulated in response to SARS-CoV-2 infection. More research is needed to highlight which of the UPS-related changes that these authors observed are due to the viral manipulation of the UPS by SARS-CoV-2, or to a host response against the virus [109].

##### ERAD/UPS-Related SARS-CoV-2 Strategies

SARS-CoV-2 can manipulate the host ERAD/UPS systems or introduce viral UPS components (Figure 2). The reported ERAD and UPS components hijacked by SARS-CoV-2 are summarized in Table 2.

Multiomics studies have shown that SARS-CoV-2 proteins undergo ubiquitination, facilitating viral replication, and some E3 ubiquitin ligases have been identified as potential antiviral targets [110]. In addition, Longhitano et al. (2020) discussed the potential use of UPS inhibitors for therapeutic interventions, reducing viral entry, RNA synthesis, and protein expression [111]. A comprehensive overview of the roles of E3 ubiquitin ligases and DUBs in COVID-19 has recently been presented by Zhao et al. (2023). They clarified the mechanisms by which the virus utilizes host E3 ubiquitin ligases and DUBs, along with its own viral proteins that have similar enzyme activities, to facilitate invasion, replication, escape, and inflammation [73]. Several authors have demonstrated that ubiquitin variants (UbVs) can block SARS-CoV-2 PLpro activity, providing novel insights into drug development [63,112]. These discoveries highlight the virus’ remarkable ability to hijack host cellular machinery to further its own replication and propagation. Besides this, Chen et al. (2022) have suggested that SARS-CoV-2 may sequester the ERAD pathway and EDEMosome formation machinery, similar to other coronaviruses [113]. This is supported by the fact that the SARS-CoV-2 ORF8 protein induces an increase in EDEM expression in 293-F cells, derived from transformed human embryonal kidney (HEK) cells, but not in mouse cells. Due to the context-specific interaction of the virus and the host cells, further detailed studies are required to precisely elucidate the virus’ specific role in ERAD during the course of infection. Maimaitiyiming et al. (2022) demonstrated that heat treatment can promote the ubiquitin-mediated proteolysis of SARS-CoV-2 RNA polymerase (NSP12), offering a potential strategy for controlling viral replication. This mechanism involves the E3 ubiquitin ligase ZNF598, which plays a crucial role in the degradation process [106]. Several authors have also proposed that DUBs could be potential therapeutic targets against SARS-CoV-2 infection [114]. For example, inhibitors of the PLpro, such as PR-619 and HBX41108 or naphthalene based PLpro inhibitors, restrict SARS-CoV-2 replication in both VERO-B4 and human Calu-3 lung cells [112,115,116]. Other studies have suggested that proteasome inhibitors, such as MG132, epoxomycin and bortezomib, negatively impact the replication of various viruses, including SARS-CoV-2, within host cells [111].

**Table 2:** Summary of reported ERAD and UPS components hijacked by SARS-CoV-2.

	Key Components	SARS-CoV-2 Strategies	References
**ERAD and UPS**	Ubiquitination	Facilitates viral replication of SARS-CoV-2 proteins.	[105]
	E3 Ubiquitin Ligases and DUBs	Identified as potential antiviral targets. Utilized by the virus for invasion, replication, escape, and inflammation.	[68,105]
	Ubiquitin Variants (UbVs)	Block SARS-CoV-2 PLpro activity, offering insights for drug development.	[58,107]
	Heat Treatment	Promotes ubiquitin-mediated proteolysis of SARS-CoV-2 RNA polymerase (NSP12) through E3 ubiquitin ligase ZNF598.	[101]
	DUBs Inhibitors	Potential therapeutic targets against SARS-CoV-2 infection.	[107,109,110,111]
	Proteasome Inhibitors	MG132, epoxomycin, and bortezomib negatively impact SARS-CoV-2 replication.	[106,107]

In conclusion, the interaction between SARS-CoV-2 and the ERAD/UPS intricately shapes SARS-CoV-2 infection dynamics. Understanding these mechanisms would provide valuable insights into potential therapies for combatting COVID-19-induced neurodegeneration.

### 2.3. Autophagy

Autophagy is a fundamental cellular process responsible for the degradation and recycling of cellular components, playing a crucial role in maintaining cellular homeostasis by removing damaged organelles and cellular debris, protein aggregates and specific soluble proteins [117,118]. There are three main types of autophagy: macroautophagy, microautophagy, and chaperone-mediated autophagy (CMA) (Figure 3). All of them culminate in the delivery of the target substrate to the lysosome for degradation. Macroautophagy involves the formation of autophagosomes that engulf cytoplasmic material, which then fuse with lysosomes [119]. Microautophagy directly engulfs cargo at the lysosomal membrane [120], while CMA selectively targets proteins recognized by specific chaperone proteins [121]. Each type of autophagy is orchestrated by a distinct set of key proteins [118]. In macroautophagy, initiation involves the contribution of unc-51-like autophagy-activating kinases 1 and 2 (ULK1/2), autophagy-relate gen 13 (ATG13), FAK family-interacting protein of 200 kDa (FIP200), and ATG101, while autophagosome nucleation relies on Beclin-1 (BECN1), vacuolar protein sorting 34 (VPS34), VPS15, and ATG14. Elongation requires ATG5, the ATG12–ATG5–ATG16L1 complex, ATG7, ATG10, and ATG3. The microtubule-associated protein 1A/1B-light chain 3 (LC3), the gamma-aminobutyric acid receptor-associated protein (GABARAP), the lysosomal-associated membrane protein 2 (LAMP2) and the sequestosome-1 (SQSTM1/p62, hereafter referred to as p62) participate in the recognition of cargo, and autophagosome maturation and fusion [119,122]. Microautophagy involves the endosomal sorting complexes required for transport (ESCRT) machinery and heat-shock cognate 70 (HSC70) in endosomal microautophagy, and LAMP2A and HSC70 in lysosomal microautophagy [120,123]. Chaperone-mediated autophagy relies on HSC70, co-chaperones, and LAMP2A, wherein KFERQ-like motifs guide substrate recognition and translocation into lysosomes [121,124]. These key proteins, together with other involved proteins, collectively govern the dynamic and selective degradation of cellular components, illustrating the intricate molecular machinery underlying the diverse autophagic pathways. Additionally, various specialized forms of autophagy have been defined, such as mitophagy (targeting damaged mitochondria), pexophagy (clearing peroxisomes), aggrephagy (removing protein aggregates), lipophagy (degrading lipid droplets), ER-phagy (eliminating parts of the ER), and nucleophagy (selective removal of nuclear components). Each targets specific cellular structures, ensuring the maintenance of cellular health through controlled degradation and recycling.

Autophagy plays a complex role in cellular health. Proper autophagic function is essential for neuronal survival, as it clears misfolded proteins associated with neurodegenerative diseases. However, dysfunctional autophagy can contribute to the accumulation of toxic protein aggregates, a hallmark of conditions like AD and PD disease, among other neurodegenerative diseases [125,126,127]. Numerous preclinical studies provide evidence supporting the potential use of autophagy modulators as suppressors of age-related pathologies, particularly in the context of neurodegenerative diseases [127]. In viral infections, similar to the ERAD/UPS systems, autophagy plays a dual role. On one hand, it serves as a crucial host defense mechanism by targeting and degrading viral components, contributing to the elimination of the virus. On the other hand, certain viruses have evolved to exploit the autophagic process for their own replication and survival, subverting the host defense mechanisms for their benefit [128,129,130]. This intricate interplay between autophagy and viral infections highlights the complexity of host–virus interactions and the multifaceted nature of cellular responses to viral invasion. As for neurodegeneration, modulating autophagic activity has emerged as a promising approach to combat viral infections [131]. 

#### 2.3.1. Autophagy in Viral Infections

On the subject of viral infections, by selectively targeting viral components, degrading replication compartments, facilitating antigen presentation, and integrating with antiviral signaling pathways, autophagy serves as a potent cellular defense mechanism against a wide range of viral infections. However, similar to what happens with the other protein quality control systems, persisting viruses have evolved strategies to manipulate the autophagic machinery [128,129,130]. The role of autophagy in viral infections has recently been reviewed by Chen et al., (2023) [128]. Many studies have demonstrated that autophagy can execute its antiviral function through different lines of attack [132]:

− The selective autophagic degradation of viral components. Autophagy can selectively target viral components, such as viral proteins and nucleic acids, for degradation. For example, selective autophagy has been observed to degrade the capsid protein of the Sindbis virus (SIN) in the CNS [133] or the p62 targeted Dengue virus (DENV) capsid protein [134]. Picornaviruses, such as poliovirus, are recognized by galectin 8, which limits viral infection by triggering the autophagic degradation of the viral RNA genome [135]. Activated antiviral autophagy in *Drosophila melanogaster* restricts ZIKV infection in the brain [136]. This targeted degradation hampers viral replication and restricts the spread of infection;
− A role in antigen presentation and adaptive immunity. Autophagy plays a crucial role in antigen presentation. Viral antigens captured by autophagosomes can be processed and presented on MHC molecules, leading to the activation of virus-specific T cells [137,138]. This process enhances the adaptive immune response against viral infections.
− A role in the innate immune response. There is an interplay between interferons and autophagy. Various antiviral signaling pathways, such as the IFN pathway, can induce autophagy in response to viral infections. This induction of autophagy is often linked to the restriction of viral replication [130,139]. At the same time, autophagy can promote interferon production, cooperating with pattern recognition receptor signaling [130,132].

These findings collectively highlight the multifaceted antiviral mechanisms employed by autophagy. On the other side of the battle, viruses have evolved mechanisms to subvert the autophagic process for their advantage. Some viruses inhibit autophagy to evade degradation, while others exploit autophagosomes for replication or use the pathway to enhance viral maturation and transmission [128,129,140]. Viruses manipulate cellular autophagy machinery at various stages of infection [128]:

− Initiation of autophagy. Measles virus (MV) induces autophagy via CD46-Cyt-1, facilitating viral entry [141], and the HCV triggers autophagy through ER stress and reactive oxygen species (ROS), crucial for replication [142]. Other viruses can inhibit autophagy initiation, like HSV-1, which employs the infected cell protein 34.5 (ICP34.5) to interact with BECN1, impairing autophagy initiation [143]. Also, the HCMV utilizes BCL-2 and the viral proteins IRS1 and TRS1 to disrupt BECN1 function, hindering autophagosome formation [144]. Similarly, the Kaposi’s sarcoma-associated herpesvirus (KSHV) expresses a virally encoded G protein-coupled receptor (vGPCR), which activates the mammalian target of rapamycin (mTOR) pathway and downregulates ATG14L expression, leading to the inhibition of autophagy initiation [145];
− Viral replication in Double-Membrane Vesicles (DMVs). Coronaviruses depend on autophagy-induced DMVs for RNA synthesis and maturation [146]. Likewise, HCV utilizes DMVs containing viral proteins as replication organelles [147];
− Autophagosome–lysosome fusion inhibition. The M2 protein of the Influenza A virus (IAV) prevents autophagosome maturation, promoting viral survival [148]. In addition, the Human parainfluenza virus type 3 (HPIV3) hinders fusion by enhancing viral particle production [149];
− Secretory autophagy for viral maturation. DENV utilizes autophagy-associated vesicles for efficient transmission, encapsulating virions in vesicles [150]. Enteroviruses (like Poliovirus) exploit autophagosome-like vesicles for cell-to-cell spread [151].

These mechanisms highlight the viruses’ ability to exploit autophagy for their survival and propagation.

In conclusion, autophagy serves as a critical cellular process in the context of viral infections, acting both as a defense mechanism and a target for viral exploitation. Further research in this field continues to uncover the intricate mechanisms underlying the relationship between autophagy and viral infections, paving the way for the development of novel antiviral therapies.

#### 2.3.2. Autophagy and SARS-CoV-2

We have also explored the literature on the interplay between autophagy and SARS-CoV-2 infection, which evidently elucidates the dual role of autophagy as a defender against viral intrusion and a facilitator of viral proliferation.

##### Antiviral Role of Autophagy in SARS-CoV-2 Infection

In the context of SARS-CoV-2 infection, autophagy, in its canonical role, acts as a surveillance system, detecting and engulfing viral particles and damaged cellular organelles into autophagosomes, as reported for other viruses in the previous paragraph. Many studies shed light on the interaction between SARS-CoV-2 infection and autophagy; however, they mainly focus on the viral manipulation of this system, which at the same time highlights the importance of autophagy as a defense mechanism in SARS-CoV-2. In spite of the lack of specific literature showing a classical role of autophagy against SARS-CoV-2, we still found some evidence. The ubiquitination of ACE2, the receptor used by SARS-CoV-2 for cell entry, is recognized by the autophagic receptor toll interacting protein (TOLLIP). This interaction facilitates the delivery of ACE2 to lysosomes for selective autophagic degradation. By reducing ACE2 availability, this process limits viral entry [152]. Li et al. (2021) demonstrated that SARS-CoV-2 induces ROS production and the consequent activation of autophagy [153]. Also, Silva et al. (2022) showed that the induction of autophagy by IFN-I inhibits SARS-CoV-2 replication in cells expressing IFN-I receptors (IFNAR1 and IFNAR2) [154]. The activation of autophagy in these cells leads to the degradation of viral components, limiting the replication of SARS-CoV-2 and contributing to the host antiviral defense mechanisms. Besides this, the pharmacological activation of autophagy inhibits SARS-CoV-2 replication [155,156,157]. Consistently, in severe cases of COVID-19, Tomic et al. (2021) observed a significant decrease in the expression of specific autophagy-related genes, including ULK1, ATG5, UV radiation resistance-associated gene protein (UVRAG), activating molecule in BECN1-regulated autophagy protein 1 (AMBRA1), PIK3C3, and LC3, in comparison to healthy individuals [158]. In opposition, other studies like that by Shang et al. (2021) showed that the inhibition of autophagy in human ACE2 transgenic mice and xenografted human lung tissues suppresses SARS-CoV-2 replication [159]. 

In VERO-E6 and Huh-7 cells, the presence of SARS-CoV-2 triggers the creation of autophagosomes by the suppression of the AKT/mTOR pathway and the activation of the ULK-1-ATG13 and VPS34-VPS15-BECN1 pathways. However, the virus disrupts the fusion between autophagosomes and lysosomes, hampering the completion of the autophagic process [159]. Moreover, the lysosomal protein LAMP2, associated with CMA, interacts with viral RNA, impairing SARS-CoV-2 replication [160]. Thus, even if the virus is able to inhibit autophagosome–lysosome fusion [161], part of the virus reaches the lysosomes, whereupon they would be degraded.

We hypothesize that after SARS-CoV-2 infection, autophagy is initiated, at least in part, as an antiviral process of the host cell, but it could also be induced as a viral strategy to produce the generation of autophagosomes for replication. However, viral clearance is only achieved when the virus is not able to control the lysosome degradation. For that, the pharmacological activation of autophagy probably exceeds the capacity of the virus to suppress autophagosome–lysosome fusion, and reduces viral replication. Likewise, autophagy inhibition would reduce viral replication as SARS-CoV-2 requires the formation of autophagosomes for its replication and egress.

##### Proviral Role of Autophagy in SARS-CoV-2 Infection

Different SARS-CoV-2 proteins orchestrates autophagy modifications at various stages and in different types [162]. Among the different types of autophagy (Figure 3), the available data are mainly related to macroautophagy, including ER-phagy and mitophagy [161,162,163].

The manipulation of autophagy by SARS-CoV-2, as well as the proteins involved (summarized in Table 3), seem to be both infection stage- and cell type-dependent [162,164]. Understanding the intricate proviral role played by autophagy, after being hijacked by the virus, in different cell types and infection stages is paramount for devising effective treatments against COVID-19. SARS-CoV-2 manipulates several phases of the autophagy system to facilitate its replication, evade immune responses, and promote its survival within host cells (Figure 3). In particular, SARS-CoV-2 is able to induce or to block autophagy and autophagosome formation, suppress autophagosome–lysosome fusion, and induce its extracellular release with the help of autophagy to achieve the different infection steps [161,162,164,165,166,167] (Figure 3). Regarding the early steps of macroautophagy, SARS-CoV-2 can block phagophore and autophagosome formation, mostly from the ER membrane (ER-phagy), through the NSP3, NSP6, NSP15, ORF3a and ORF8 viral proteins, by reducing the lipidation of LC3, promoting the degradation of BECN1, disrupting the formation of the initiation complex of ULK1 and ATG13, or by sequestering the ER-phagy receptors FAM134B and ATL3 into p62 condensates [161,162,163,168,169,170]. However, NSP4, NSP6, ORF8 and NSP13 have a positive regulatory effect on autophagosome formation [161,162,171,172,173]. In the case of NSP6, the sizes of autophagosomes are smaller than those induced by starvation, and this is related to the formation of DMVs as viral replication compartments [162]. Furthermore, the suppression of autophagy initiators like ATG5, ATG7, BECN1, and FIP200 by small interfering RNA (siRNAs) reduces SARS-CoV-2 replication in vitro [155]. Even if SARS-CoV-2 promotes autophagosome accumulation, viral proteins like NSP3, NSP6, ORF3a, ORF7a, M, and E can also block the later steps of autophagy (autophagosome–lysosome fusion, autolysosome acidification and degradation), for example by interacting with VPS39 and UVRAG, and disrupting the soluble N-ethylmaleimide-sensitive factor activating protein receptor (SNARE) and the homotypic fusion and vacuole protein sorting (HOPS) complexes [128,161,162,174,175,176,177]. Finally, after replicating, SARS-CoV-2 undergoes secretion and release by promoting the fusion of SARS-CoV-2-containing vesicles and lysosomes with the plasma membrane, for the transmission of the infection to non-infected cells [113,161,164,165]. In addition to promoting viral replication and release, the manipulation of autophagy by SARS-CoV-2 leads to immune evasion. On the one hand, viral proteins such as ORF10 impair mitophagy by blocking the interaction between p62 and LC3, leading to mitochondrial dysfunction and weakened immune responses [162,178,179]. On the other hand, SARS-CoV-2 utilizes the ORF8 protein to hijack the autophagy pathway, leading to the downregulation of MHC-I molecules [166]. In COVID-19 patients, diminished T cell activation may stem from impaired antigen presentation and the altered expression of autophagy-related genes [158,164]. This process shields infected cells from recognition and eradication by T cells, enhancing the virus’ ability to persist within the host organism. Furthermore, SARS-CoV-2 can also inhibit the cGAS-STING-mediated autophagy flux, suppressing antiviral responses [180,181,182].

Regarding the interconnection between the UPS and autophagy in SARS-CoV-2 infection, specific viral proteins, like the membrane (M) protein, are K63-ubiquitinated, guiding them to autophagosomes for degradation, and thus limiting viral spread. Certain SARS-CoV-2 proteins, including 3C-like protease (3CL), ORF9b, NSP8, and PLpro, modify this ubiquitination, influencing autophagy and aiding viral replication [183,184]. Understanding this interaction is vital, revealing the virus’ manipulation of host cells and highlighting potential therapeutic targets. Further research is crucial to derive detailed insights and antiviral strategies.

These viral strategies, coupled with the disruption of the host’s innate immune responses, enhance viral replication and dissemination. Continued research is essential to unravel the precise mechanisms and pathologies associated with autophagy in the context of COVID-19. Such insights are pivotal for the development of targeted and effective therapeutic strategies against COVID-19.

**Table 3:** Summary of reported autophagic components hijacked by SARS-CoV-2.

	Key Components		SARS-CoV-2 Strategies	References
**Autophagy**	Early Autophagy Steps	NSP3, NSP6, NSP15, ORF3a, ORF8	Block phagophore and autophagosome formation from the ER, but positive regulatory effect on autophagosome formation in general; smaller autophagosomes (related to DMVs).	[156,157,158,163,164,165,166,167,168]
	Later Autophagy Steps	NSP3, NSP6, ORF3a, ORF7a, M, E	Block autophagosome–lysosome fusion and autolysosome acidification and degradation.	[123,156,157,169,170,171,172]
	Autophagy and Immune Evasion	ORF10, ORF8	Impair mitophagy; downregulation of MHC-I molecules; inhibition of cGAS-STING-mediated autophagy flux.	[157,161,173,174,175,176,177]
	UPS and Autophagy Interaction	M protein	K63-ubiquitination of viral proteins for autophagic degradation.	[178,179]
	Autophagy Inhibition	ATG5, ATG7, BECN1, FIP200	Suppression reduces SARS-CoV-2 replication.	[150]

### 2.4. Molecular Chaperones

Molecular chaperones are crucial cellular components for the maintenance of proteostasis. They are responsible for assisting in the proper folding of proteins, preventing misfolding, and aiding in the assembly and transport of proteins to their functional destinations [185]. These chaperones are localized in different cellular compartments, reflecting their diverse roles in assisting protein folding within specific environments. The major cellular locations for molecular chaperones include the cytoplasm (such as the heat shock proteins (HSPs) HSP70, HSP90 and Chaperonin/HSP60 families), ER (GRP78, GRP94, calnexin and calreticulin), mitochondria (HSP70 and HSP60), and the nucleus (HSP70 and HSP90) [186]. Their functional roles extend beyond their involvement in normal cellular functions; they play a critical role in various pathological conditions. In the context of neurodegenerative diseases, such as AD and PD, the misfolding and aggregation of specific proteins like tau and α-synuclein are associated with chaperone dysfunction [187,188,189]. Chaperones like HSP27, HSP70 and HSP90, which are typically involved in preventing protein aggregation, may become overwhelmed or less effective, contributing to the progression of these diseases [190,191]. On the other hand, viral infections can retain the host chaperones, prompting a kind of chaperone dysfunction due to the alterations in the chaperone activity and target substrates to be folded, induced by the virus [192,193,194]. Again, the viruses can perturb the protein quality control systems, probably facilitating the neurodegenerative process.

#### 2.4.1. Molecular Chaperones in Viral Infections

In the context of viral infections, the overexpression of specific chaperones can potentially lead to antiviral effects by stimulating immune responses against the virus and promoting the death of infected cells, as is supported by many studies [193,195,196,197]. The HSPs are crucial in inhibiting viral proliferation by interacting with viral molecules [198]. For example, HSC70/HSP90 helps assemble the RNA-induced silencing complex (RISC), repressing viral translation [199]. In HPV, the secreted HSP70 boosts immune responses [200]. The HSPs and small HSPs also regulate immune pathways; HSP60 influences cellular immunity, HSP40 disrupts MDA5 multimer formation, and upregulated HSP27 inhibits virus replication via nuclear factor-kappa B (NF-κB) activation [201,202,203]. Even though the molecular chaperones are key to antiviral defense by modulating immune responses and interacting with viral components, viruses lacking their own chaperones rely on the host chaperone proteins for their survival and replication [192,193]. Many molecular chaperones, including HSP40, HSP60, HSC/HSP70, HSP90, GRP78, calnexin, calreticulin, and the chaperonin containing tailless complex (CCT), have been highlighted for their significant roles in the life cycles of diverse viruses [194]. For example, HSP40 assists HIV-1 in enhancing viral gene expression and replication, while also aiding IAV in the nuclear import of viral ribonucleoprotein [194,204,205]. HSP60 participates in HBV polymerase activation, replication initiation, and virus-mediated apoptosis [196], and also promotes apoptosis in HCV-infected cells [206]. Those from the HSP70 and HSP90 families help fold their proteins and improve their survival in the host [198,207]. These HSPs assist in the replication of various viruses and directly interact with viral enzymes, enhancing viral replication. Specifically, HSC/HSP70 acts as a host–cell membrane receptor for Rotaviruses, DENV and JEV, promoting virus entry, infectivity, and replication [208,209]. HSP70 can interact with viral polymerase, favoring replication, while HSP90 is crucial for the expression of viral genes. Both HSP70 and HSP90 can team up and facilitate the virus’ entry into host cells [210]. HSP90 facilitates DENV and IAV entry, positively regulates HCV replication, and mediates EBV DNA polymerase assembly [208,211,212].

Other type of molecular chaperones, like ER, nuclear and mitochondrial chaperones, can also be hijacked by viruses [213]. For instance, in HIV infections, mitochondrial HSP70 and DNAJB1 play a crucial role in stimulating transcription and replication. They achieve this by stabilizing the viral Nef protein [204,214,215]. Furthermore, Simian Virus 40 (SV40) and HCMV employ ER chaperones like GRP78, HSC70 and HSP105 for virus stabilization and immune evasion, underscoring their sophisticated strategies for proliferation within host cells [92,216]. Besides this, GRP78 has been identified as a key player in ZIKV binding, internalization, and replication within cells [217]. Under viral infections, GRP78 can undergo translocation to the cell membrane, transforming into cell surface GRP78 (CS-GRP78), which plays a crucial role in facilitating viral entry by enhancing attachment and improving the virus’ ability to enter host cells. This phenomenon is observed in various viral infections, including the MERs-CoV, the bat coronavirus HKU9 and DENV [19,218,219]. GRP78 also regulates CMV virion assembly and blocks apoptosis in HCV-infected cells [220]. Moreover, the ER chaperones calnexin and calreticulin ensure the proper assembly and maturation of Rotavirus particles [221]. Additionally, the CCT assists IAV, Rabies virus, DENV, and Reovirus in promoting virus replication, transcription, and viral protein folding [222,223,224]. Another type of molecular chaperone is the prefoldin, which is also crucial to specific viral infections [225]. In cases of Reovirus, they aid in capsid protein folding, promoting viral assembly [226]. The HCV frameshift (F) protein disrupts prefoldin function, impairing HCV replication [227]. Additionally, prefoldin subunit 3 (PFDN3) mediates HIV-1 integrase degradation, enhancing viral gene transcription and replication [228].

In summary, molecular chaperones serve as critical players in the sophisticated interplay between viruses and host cells, influencing viral entry, replication, and overall infection dynamics.

#### 2.4.2. Molecular Chaperones and SARS-CoV-2 Infection

There has been extensive research on the interaction of SARS-CoV-2 with molecular chaperones, although the literature is mainly focused on the proviral role of this interaction. The molecular chaperones manipulated by SARS-CoV-2 are summarized in Table 4. Recent studies have revealed the essential role of host HSP90 in human coronaviruses such as MERS-CoV, SARS-CoV, and SARS-CoV-2 [198,210,229]. The reliance of human coronaviruses on HSP90 underscores a potential antiviral target. In fact, several inhibitors of HSP90, such as 17-AAG, 17-DMAG or Luminespib, exhibit antiviral properties against SARS-CoV-2. They not only impede viral replication, but also suppress inflammatory responses, including the downregulation of pro-inflammatory cytokines such as interleukin 6 (IL-6), CXCL10, and CXCL11 [198,229,230]. Similarly, the transcriptomic profile of human cell lines after infection with SARS-CoV-2, as outlined by Wyler et al., (2021), identifies HSP90 as a target for COVID-19 therapy [231]. HSP90, as well as HSP70, are crucial for viral gene expression, assisting in capsid assembly [230]. On the other hand, the ER chaperone GRP78 is upregulated during SARS-CoV-2 infection, mainly due to the virus-induced ER stress, and acts as a pro-viral protein [48,232,233]. The virus relies on GRP78 for its entry into host cells and subsequent viral protein production. The knockdown or inhibition of GRP78 using siRNA, specific inhibitors like HA15, or GRP78-depleting antibodies, blocks viral entry and infection both in vitro and in vivo [234,235]. It has been recently demonstrated that inhibiting GRP78 with YUM70 suppresses the entry of SARS-CoV-2 into cells, decreases the production of spike proteins, and alleviates lung damage caused by the virus [236]. The interplay between molecular chaperones and SARS-CoV-2 infection underscores their dual roles, with HSP90 and GRP78 inhibitors showing potential as antiviral agents. Further research is needed to explore the involvement of other chaperones and their potential as therapeutic targets against COVID-19.

**Table 4:** Summary of reported molecular chaperones hijacked by SARS-CoV-2.

	Key Components	SARS-CoV-2 Strategies	References
**Molecular Chaperones**	**HSP90**	Essential for SARS-CoV-2 life cycle. HSP90 facilitates the correct folding and assembly of viral proteins, ensuring the proper formation of functional viral particles.	[193,224]
	**HSP90 inhibition**	17-AAG, 17-DMAG and Luminespib suppress SARS-CoV-2 replication. Reduction in pro-inflammatory cytokines modulating the host immune response during SARS-CoV-2 infection.	
	**HSP70**	Crucial for SARS-CoV-2 gene expression, assists in capsid assembly.	[225]
	**GRP78**	Upregulated during SARS-CoV-2 infection, acts as a pro-viral protein.	[45,227,228,229,230,231]

## 3. Cell-Specific Effects of SARS-CoV-2 in the Brain

The question of whether SARS-CoV-2 can enter the CNS has been a matter of debate [237,238], but most of the recent evidence suggests that the virus can enter the CNS, triggering inflammatory responses and causing direct cell damage [239]. Proposed entry routes include the olfactory nerve and potential breach of the blood–brain barrier [240]. Some authors have suggested that the SARS-CoV-2 virus may use the nervus terminalis rather than the olfactory nerve as a shortcut from the nasal cavity to infect the brain [241]. However, the exact mechanisms and clinical significance of SARS-CoV-2 infection of the CNS are not yet fully understood. It has been shown that SARS-CoV-2 is associated with changes in brain structure and increase the permeability of the blood–brain barrier (BBB) [237,242]. A recent study by Villadiego et al. (2023) shows that SARS-CoV-2 is not able to infect all cell types in the brain [239,240]. The virus seems to predominantly infect the neurons, but not astrocytes, and the colocalization of SARS-CoV-2 and microglial cells may correspond to viral particles originating from infected neurons or damaged vascular cells that have been phagocyted by microglial cells [240]. However, not only ACE2, but also receptors like CD147, TMPRSS2, and NRP1, have been identified in brain cells, as potential facilitators of SARS-CoV-2’s entry into the CNS. Further research is required to elucidate the cell-specific interplay between SARS-CoV-2 and the different brain cells, particularly concerning alterations in protein quality control systems induced by the virus.

Interestingly, when comparing cell dependence on proteostasis, studies demonstrate that neurons but not glial cells are particularly susceptible to disruptions in protein homeostasis [243]. For instance, disrupting autophagy in microglial cells does not induce microglial degeneration, underscoring their resilience [244,245]. Additionally, glial cells present higher proteasome activity compared to neurons, and they are more efficient in maintaining cellular protein homeostasis than neurons [246]. Notably, neurodegeneration is primarily associated with neuronal death rather than the death of microglial cells or astrocytes. In many neurodegenerative disorders, such as AD, PD, or Huntington’s disease, the hallmark feature is the progressive loss of neurons in specific regions of the brain [247]. Considering that SARS-CoV-2 infection predominantly targets neurons rather than glial cells, this could explain why disruptions of the protein quality control systems induced by SARS-CoV-2 lead to significant neurodegeneration [248,249,250].

## 4. Link between SARS-CoV-2, ER Stress and Neurodegeneration

In addition, accumulating evidence has demonstrated an association of viruses with neurodegenerative disorders [251,252,253,254,255,256]. This association involves virus-induced neuroinflammation, disruptions in the protein quality control systems and the induction of oxidative stress, with consequent protein aggregation [257,258,259,260,261]. SARS-CoV-2 has been associated with various short- and long-term neurological manifestations, ranging from mild symptoms to severe complications [6,7,8,262,263]. Similarly to other viruses, SARS-CoV-2 can exacerbate existing neurodegenerative conditions or contribute to their development through mechanisms such as neuroinflammation, protein aggregation, ER stress and oxidative stress [42,164,240,261,264,265,266,267,268,269,270,271]. For instance, recent studies reveal SARS-CoV-2 targeting cortical neurons, inducing MAPT/tau pathologies and neurodegeneration [164,272].

A study by Prasad et al. (2022) employed a sophisticated approach called “systems biology-based network analysis” to understand the genetic associations between COVID-19 and various brain disorders [273]. They identified the UPS and ERAD as the main altered mechanisms associated with SARS-CoV-2-induced neurological disorders. Furthermore, evidence from postmortem studies indicates that the SARS-CoV-2 components detected in the frontal lobe may be associated with intracytoplasmic vesicles related to the autophagic–lysosomal pathway [274]. In fact, the systemic inflammation induced by COVID-19 may impact brain autophagy, as pro-inflammatory cytokines like TNF can drive glial polarization toward the M1 phenotype, blocking the autophagy pathway and contributing to neuroinflammation [164,275]. The viral blockage of autophagosome–lysosome fusion, and autolysosome acidification and degradation, impedes the degradation of protein aggregates, and worsens ER stress and protein accumulation [128,161,162,174,175,176,177], which are even more impaired by the manipulation of the molecular chaperones previously described [48,230,231,232,233,234,235,236].

The defects in the protein quality control systems induced by SARS-CoV-2 result in prolonged ER stress. Under persistent ER stress, the UPR transitions from pro-survival to pro-apoptotic responses [276,277]. This shift involves the initiation of pro-apoptotic signaling pathways (Figure 1) (e.g., IRE1//TRAF2/JNK/MAPK or PERK/ATF4/CHOP), as well as activated p53 and BAX/BAK caspase cell death, disturbances in calcium levels due to ER release, ROS generation and oxidative stress, and mitochondrial dysfunction [14]. The intricate interplay of these factors highlights the multifaceted nature of ER stress-induced cell death pathways.

Oxidative stress, in particular, contributes to a detrimental feedback loop, exacerbating ER stress and prompting cell death.

A redox imbalance is implicated in the pathogenesis of COVID-19, potentially triggering mitochondrial dysfunction and the production of proinflammatory cytokines. The SARS-CoV-2 virus activates Toll-like receptors (TLRs), particularly TLR4, initiating the NF-κB pathway. This activation triggers the release of proinflammatory cytokines such as IL-1, IL-2, IL-6, IL-8, IL-12, and TNFα, resulting in a cytokine storm. The excessive inflammation, characteristic of severe COVID-19, stimulates the overproduction of ROS, including superoxide anion radicals, which further induces the activation of the NF–κB pathway [267,278,279].

In the brain, the generation of ROS contributes to ongoing damage by disrupting the cellular antioxidant regulatory system, but also protein folding, inducing ER stress [261,280]. Defective mitochondria also generate aberrant amounts of ROS, causing more oxidative stress and disturbing cellular homeostasis due to the disruption of the balance between ROS generation and antioxidant function [281,282]. ROS also activates various enzymes, such as myeloperoxidase, xanthine oxidase and NADPH oxidase (NOX2), and reduces the activity of glutathione peroxidase, further amplifying ROS production.

Additionally, SARS-CoV-2-induced iron dysregulation contributes to ROS generation, fostering the pro-oxidative environment, ER stress, and the ensuing cell death [267]. The resulting imbalance in ROS homeostasis induces neuroinflammation, demyelination, and axonal damage, resembling mechanisms observed in neurodegenerative disorders like AD and PD. Moreover, SARS-CoV-2 induces the upregulation of cyclooxygenase-2 (COX-2), high-mobility group box protein 1 (HMGB1), and the receptor for advanced glycation end-products (RAGE). These biomolecules are associated with hyperinflammation and high levels of ROS, thus serving as potential indicators of severe cases of COVID-19 and neurodegenerative processes [283].

In summary, SARS-CoV-2 infection seems to produce neurodegeneration through the induction of ER stress, oxidative stress, neuroinflammation, and the impairment of protein quality control systems. After reviewing the current literature, we hypothesize a direct connection between alterations to protein quality control systems and neurodegeneration after SARS-CoV-2 infection. However, further research, including longitudinal studies, will be essential to unravelling the intricate relationship between SARS-CoV-2 and neurodegeneration.

## 5. Conclusions and Future Directions

SARS-CoV-2 inducespersistent ER stress, which cannot be resolved by the host’s cells due to the viral hijacking of the protein quality control systems. The consequent proteostasis imbalance, together with the induced oxidative stress and neuroinflammation, lead to neurodegeneration. This review encourages more research on this potential link to bring about novel treatment modalities for this area of clinical urgency.

## References

[1] Jackson C.B., Farzan M., Chen B., Choe H. (2022). Mechanisms of SARS-CoV-2 entry into cells. Nat. Rev. Mol. Cell Biol..

[2] Kakavandi S., Zare I., VaezJalali M., Dadashi M., Azarian M., Akbari A., Ramezani Farani M., Zalpoor H., Hajikhani B. (2023). Structural and non-structural proteins in SARS-CoV-2: Potential aspects to COVID-19 treatment or prevention of progression of related diseases. Cell Commun. Signal..

[3] Benton D.J., Wrobel A.G., Xu P., Roustan C., Martin S.R., Rosenthal P.B., Skehel J.J., Gamblin S.J. (2020). Receptor binding and priming of the spike protein of SARS-CoV-2 for membrane fusion. Nature.

[4] Barnes C.O., Jette C.A., Abernathy M.E., Dam K.A., Esswein S.R., Gristick H.B., Malyutin A.G., Sharaf N.G., Huey-Tubman K.E., Lee Y.E. (2020). SARS-CoV-2 neutralizing antibody structures inform therapeutic strategies. Nature.

[5] Yang H., Rao Z. (2021). Structural biology of SARS-CoV-2 and implications for therapeutic development. Nat. Rev. Microbiol..

[6] Harapan B.N., Yoo H.J. (2021). Neurological symptoms, manifestations, and complications associated with severe acute respiratory syndrome coronavirus 2 (SARS-CoV-2) and coronavirus disease 19 (COVID-19). J. Neurol..

[7] Maiese A., Manetti A.C., Bosetti C., Del Duca F., La Russa R., Frati P., Di Paolo M., Turillazzi E., Fineschi V. (2021). SARS-CoV-2 and the brain: A review of the current knowledge on neuropathology in COVID-19. Brain Pathol..

[8] Rogers-Brown J.S., Wanga V., Okoro C., Brozowsky D., Evans A., Hopwood D., Cope J.R., Jackson B.R., Bushman D., Hernandez-Romieu A.C. (2021). Outcomes Among Patients Referred to Outpatient Rehabilitation Clinics After COVID-19 diagnosis—United States, January 2020–March 2021. MMWR Morb. Mortal. Wkly. Rep..

[9] Jha N.K., Ojha S., Jha S.K., Dureja H., Singh S.K., Shukla S.D., Chellappan D.K., Gupta G., Bhardwaj S., Kumar N. (2021). Evidence of Coronavirus (CoV) Pathogenesis and Emerging Pathogen SARS-CoV-2 in the Nervous System: A Review on Neurological Impairments and Manifestations. J. Mol. Neurosci..

[10] Villa C., Rivellini E., Lavitrano M., Combi R. (2022). Can SARS-CoV-2 Infection Exacerbate Alzheimer’s Disease? An Overview of Shared Risk Factors and Pathogenetic Mechanisms. J. Pers. Med..

[11] Boura I., Qamar M.A., Daddoveri F., Leta V., Poplawska-Domaszewicz K., Falup-Pecurariu C., Ray Chaudhuri K. (2023). SARS-CoV-2 and Parkinson’s Disease: A Review of Where We Are Now. Biomedicines.

[12] Al-Kuraishy H.M., Al-Gareeb A.I., Kaushik A., Kujawska M., Ahmed E.A., Batiha G.E. (2023). SARS-CoV-2 infection and Parkinson’s disease: Possible links and perspectives. J. Neurosci. Res..

[13] Ruano D. (2021). Proteostasis Dysfunction in Aged Mammalian Cells. The Stressful Role of Inflammation. Front. Mol. Biosci..

[14] Ghemrawi R., Khair M. (2020). Endoplasmic Reticulum Stress and Unfolded Protein Response in Neurodegenerative Diseases. Int. J. Mol. Sci..

[15] Hetz C., Saxena S. (2017). ER stress and the unfolded protein response in neurodegeneration. Nat. Rev. Neurol..

[16] Uddin M.S., Yu W.S., Lim L.W. (2021). Exploring ER stress response in cellular aging and neuroinflammation in Alzheimer’s disease. Ageing Res. Rev..

[17] Cirone M. (2021). ER Stress, UPR Activation and the Inflammatory Response to Viral Infection. Viruses.

[18] Gavilan M.P., Pintado C., Gavilan E., Jimenez S., Rios R.M., Vitorica J., Castano A., Ruano D. (2009). Dysfunction of the unfolded protein response increases neurodegeneration in aged rat hippocampus following proteasome inhibition. Aging Cell.

[19] Suaya M., Sanchez G.M., Vila A., Amante A., Cotarelo M., Garcia Carrillo M., Blaustein M. (2022). Live and let die: Signaling AKTivation and UPRegulation dynamics in SARS-CoVs infection and cancer. Cell Death Dis..

[20] Walter P., Ron D. (2011). The unfolded protein response: From stress pathway to homeostatic regulation. Science.

[21] Ron D., Walter P. (2007). Signal integration in the endoplasmic reticulum unfolded protein response. Nat. Rev. Mol. Cell Biol..

[22] Hetz C. (2012). The unfolded protein response: Controlling cell fate decisions under ER stress and beyond. Nat. Rev. Mol. Cell Biol..

[23] Gomora-Garcia J.C., Geronimo-Olvera C., Perez-Martinez X., Massieu L. (2021). IRE1α RIDD activity induced under ER stress drives neuronal death by the degradation of *14-3-3* θ mRNA in cortical neurons during glucose deprivation. Cell Death Discov..

[24] Tsuru A., Imai Y., Saito M., Kohno K. (2016). Novel mechanism of enhancing IRE1α-XBP1 signalling via the PERK-ATF4 pathway. Sci. Rep..

[25] Hetz C., Bernasconi P., Fisher J., Lee A.H., Bassik M.C., Antonsson B., Brandt G.S., Iwakoshi N.N., Schinzel A., Glimcher L.H. (2006). Proapoptotic BAX and BAK modulate the unfolded protein response by a direct interaction with IRE1α. Science.

[26] Inoue T., Tsai B. (2013). How viruses use the endoplasmic reticulum for entry, replication, and assembly. Cold Spring Harb. Perspect. Biol..

[27] Choi J.A., Song C.H. (2019). Insights into the Role of Endoplasmic Reticulum Stress in Infectious Diseases. Front. Immunol..

[28] Li S., Kong L., Yu X. (2015). The expanding roles of endoplasmic reticulum stress in virus replication and pathogenesis. Crit. Rev. Microbiol..

[29] Prasad V., Greber U.F. (2021). The endoplasmic reticulum unfolded protein response—Homeostasis, cell death and evolution in virus infections. FEMS Microbiol. Rev..

[30] Santerre M., Arjona S.P., Allen C.N., Shcherbik N., Sawaya B.E. (2021). Why do SARS-CoV-2 NSPs rush to the ER?. J. Neurol..

[31] Catanzaro N., Meng X.J. (2020). Induction of the unfolded protein response (UPR) suppresses porcine reproductive and respiratory syndrome virus (PRRSV) replication. Virus Res..

[32] Zhang L., Wang A. (2012). Virus-induced ER stress and the unfolded protein response. Front. Plant Sci..

[33] Rios-Ocampo W.A., Navas M.C., Buist-Homan M., Faber K.N., Daemen T., Moshage H. (2020). Hepatitis C Virus Proteins Core and NS5A Are Highly Sensitive to Oxidative Stress-Induced Degradation after eIF2α/ATF4 Pathway Activation. Viruses.

[34] Kolpikova E.P., Tronco A.R., Hartigh A.B.D., Jackson K.J., Iwawaki T., Fink S.L. (2020). IRE1α Promotes Zika Virus Infection via XBP1. Viruses.

[35] Johnston B.P., McCormick C. (2019). Herpesviruses and the Unfolded Protein Response. Viruses.

[36] Fang P., Tian L., Zhang H., Xia S., Ding T., Zhu X., Zhang J., Ren J., Fang L., Xiao S. (2022). Induction and modulation of the unfolded protein response during porcine deltacoronavirus infection. Vet. Microbiol..

[37] Banerjee A., Czinn S.J., Reiter R.J., Blanchard T.G. (2020). Crosstalk between endoplasmic reticulum stress and anti-viral activities: A novel therapeutic target for COVID-19. Life Sci..

[38] Yiang G.T., Wu C.C., Lu C.L., Hu W.C., Tsai Y.J., Huang Y.M., Su W.L., Lu K.C. (2023). Endoplasmic Reticulum Stress in Elderly Patients with COVID-19: Potential of Melatonin Treatment. Viruses.

[39] Wang X., Wang W., Wang T., Wang J., Jiang Y., Wang X., Qiu Z., Feng N., Sun W., Li C. (2023). SARS-CoV-2 ORF8 Protein Induces Endoplasmic Reticulum Stress-like Responses and Facilitates Virus Replication by Triggering Calnexin: An Unbiased Study. J. Virol..

[40] Liu X., Wen Y.Z., Huang Z.L., Shen X., Wang J.H., Luo Y.H., Chen W.X., Lun Z.R., Li H.B., Qu L.H. (2022). SARS-CoV-2 causes a significant stress response mediated by small RNAs in the blood of COVID-19 patients. Mol. Ther. Nucleic Acids.

[41] Bartolini D., Stabile A.M., Vacca C., Pistilli A., Rende M., Gioiello A., Cruciani G., Galli F. (2022). Endoplasmic reticulum stress and NF-kB activation in SARS-CoV-2 infected cells and their response to antiviral therapy. IUBMB Life.

[42] Chaudhry Z.L., Gamal M., Ferhati I., Warda M., Ahmed B.Y. (2022). ER Stress in COVID-19 and Parkinson’s Disease: In Vitro and In Silico Evidences. Brain Sci..

[43] Echavarria-Consuegra L., Cook G.M., Busnadiego I., Lefevre C., Keep S., Brown K., Doyle N., Dowgier G., Franaszek K., Moore N.A. (2021). Manipulation of the unfolded protein response: A pharmacological strategy against coronavirus infection. PLoS Pathog..

[44] Rashid F., Dzakah E.E., Wang H., Tang S. (2021). The ORF8 protein of SARS-CoV-2 induced endoplasmic reticulum stress and mediated immune evasion by antagonizing production of interferon beta. Virus Res..

[45] Su W.Q., Yu X.J., Zhou C.M. (2021). SARS-CoV-2 ORF3a Induces Incomplete Autophagy via the Unfolded Protein Response. Viruses.

[46] Xue M., Feng L. (2021). The Role of Unfolded Protein Response in Coronavirus Infection and Its Implications for Drug Design. Front. Microbiol..

[47] Rozpedek-Kaminska W., Siwecka N., Wawrzynkiewicz A., Wojtczak R., Pytel D., Diehl J.A., Majsterek I. (2020). The PERK-Dependent Molecular Mechanisms as a Novel Therapeutic Target for Neurodegenerative Diseases. Int. J. Mol. Sci..

[48] Shaban M.S., Muller C., Mayr-Buro C., Weiser H., Meier-Soelch J., Albert B.V., Weber A., Linne U., Hain T., Babayev I. (2021). Multi-level inhibition of coronavirus replication by chemical ER stress. Nat. Commun..

[49] Vembar S.S., Brodsky J.L. (2008). One step at a time: Endoplasmic reticulum-associated degradation. Nat. Rev. Mol. Cell Biol..

[50] Olivari S., Molinari M. (2007). Glycoprotein folding and the role of EDEM1, EDEM2 and EDEM3 in degradation of folding-defective glycoproteins. FEBS Lett..

[51] Morito D., Nagata K. (2015). Pathogenic Hijacking of ER-Associated Degradation: Is ERAD Flexible?. Mol. Cell.

[52] Jeong H., Hong E.H., Ahn J.H., Cho J., Jeong J.H., Kim C.W., Yoon B.I., Koo J.H., Park Y.Y., Yang Y.M. (2023). ERdj5 protects goblet cells from endoplasmic reticulum stress-mediated apoptosis under inflammatory conditions. Exp. Mol. Med..

[53] Lopata A., Kniss A., Lohr F., Rogov V.V., Dotsch V. (2020). Ubiquitination in the ERAD Process. Int. J. Mol. Sci..

[54] Ohtake F., Saeki Y., Ishido S., Kanno J., Tanaka K. (2016). The K48-K63 Branched Ubiquitin Chain Regulates NF-κB Signaling. Mol. Cell.

[55] Collins G.A., Goldberg A.L. (2017). The Logic of the 26S Proteasome. Cell.

[56] Trulsson F., Akimov V., Robu M., van Overbeek N., Berrocal D.A.P., Shah R.G., Cox J., Shah G.M., Blagoev B., Vertegaal A.C.O. (2022). Deubiquitinating enzymes and the proteasome regulate preferential sets of ubiquitin substrates. Nat. Commun..

[57] Paz Gavilan M., Vela J., Castano A., Ramos B., del Rio J.C., Vitorica J., Ruano D. (2006). Cellular environment facilitates protein accumulation in aged rat hippocampus. Neurobiol. Aging.

[58] Movaqar A., Yaghoubi A., Rezaee S.R., Jamehdar S.A., Soleimanpour S. (2021). Coronaviruses construct an interconnection way with ERAD and autophagy. Future Microbiol..

[59] Noack J., Bernasconi R., Molinari M. (2014). How viruses hijack the ERAD tuning machinery. J. Virol..

[60] Rao G., Croft B., Teng C., Awasthi V. (2015). Ubiquitin-Proteasome System in Neurodegenerative Disorders. J. Drug Metab. Toxicol..

[61] Dantuma N.P., Bott L.C. (2014). The ubiquitin-proteasome system in neurodegenerative diseases: Precipitating factor, yet part of the solution. Front. Mol. Neurosci..

[62] Schmidt M.F., Gan Z.Y., Komander D., Dewson G. (2021). Ubiquitin signalling in neurodegeneration: Mechanisms and therapeutic opportunities. Cell Death Differ..

[63] van Vliet V.J.E., Huynh N., Pala J., Patel A., Singer A., Slater C., Chung J., van Huizen M., Teyra J., Miersch S. (2022). Ubiquitin variants potently inhibit SARS-CoV-2 PLpro and viral replication via a novel site distal to the protease active site. PLoS Pathog..

[64] Camborde L., Planchais S., Tournier V., Jakubiec A., Drugeon G., Lacassagne E., Pflieger S., Chenon M., Jupin I. (2010). The ubiquitin-proteasome system regulates the accumulation of Turnip yellow mosaic virus RNA-dependent RNA polymerase during viral infection. Plant Cell.

[65] Fan W., Mar K.B., Sari L., Gaszek I.K., Cheng Q., Evers B.M., Shelton J.M., Wight-Carter M., Siegwart D.J., Lin M.M. (2021). TRIM7 inhibits enterovirus replication and promotes emergence of a viral variant with increased pathogenicity. Cell.

[66] Tang Q., Wu P., Chen H., Li G. (2018). Pleiotropic roles of the ubiquitin-proteasome system during viral propagation. Life Sci..

[67] Kong F., You H., Kong D., Zheng K., Tang R. (2019). The interaction of hepatitis B virus with the ubiquitin proteasome system in viral replication and associated pathogenesis. Virol. J..

[68] Choi A.G., Wong J., Marchant D., Luo H. (2013). The ubiquitin-proteasome system in positive-strand RNA virus infection. Rev. Med. Virol..

[69] Li Z., Hao P., Zhao Z., Gao W., Huan C., Li L., Chen X., Wang H., Jin N., Luo Z.Q. (2023). The E3 ligase RNF5 restricts SARS-CoV-2 replication by targeting its envelope protein for degradation. Signal Transduct. Target. Ther..

[70] Chaudhary P., Proulx J., Park I.W. (2023). Ubiquitin-protein ligase E3A (UBE3A) mediation of viral infection and human diseases. Virus Res..

[71] Rojas V.K., Park I.W. (2019). Role of the Ubiquitin Proteasome System (UPS) in the HIV-1 Life Cycle. Int. J. Mol. Sci..

[72] Voss M., Braun V., Bredow C., Kloetzel P.M., Beling A. (2021). Coxsackievirus B3 Exploits the Ubiquitin-Proteasome System to Facilitate Viral Replication. Viruses.

[73] Zhao M., Zhang M., Yang Z., Zhou Z., Huang J., Zhao B. (2023). Role of E3 ubiquitin ligases and deubiquitinating enzymes in SARS-CoV-2 infection. Front. Cell. Infect. Microbiol..

[74] Zu S., Li C., Li L., Deng Y.Q., Chen X., Luo D., Ye Q., Huang Y.J., Li X.F., Zhang R.R. (2022). TRIM22 suppresses Zika virus replication by targeting NS1 and NS3 for proteasomal degradation. Cell Biosci..

[75] Le-Trilling V.T.K., Trilling M. (2020). Ub to no good: How cytomegaloviruses exploit the ubiquitin proteasome system. Virus Res..

[76] Han K., Zhao D., Liu Y., Liu Q., Huang X., Yang J., Zhang L., Li Y. (2019). The ubiquitin-proteasome system is necessary for the replication of duck Tembusu virus. Microb. Pathog..

[77] Pang Y., Li M., Zhou Y., Liu W., Tao R., Zhang H., Xiao S., Fang L. (2021). The ubiquitin proteasome system is necessary for efficient proliferation of porcine reproductive and respiratory syndrome virus. Vet. Microbiol..

[78] Seyoum Tola F. (2023). The Role of Ubiquitin-Proteasome System in the Pathogenesis of Severe Acute Respiratory Syndrome Coronavirus-2 Disease. Int. J. Inflam..

[79] Gao W., Wang L., Ju X., Zhao S., Li Z., Su M., Xu J., Wang P., Ding Q., Lv G. (2022). The Deubiquitinase USP29 Promotes SARS-CoV-2 Virulence by Preventing Proteasome Degradation of ORF9b. mBio.

[80] Ming S.L., Zhang S., Wang Q., Zeng L., Zhou L.Y., Wang M.D., Ma Y.X., Han L.Q., Zhong K., Zhu H.S. (2022). Inhibition of USP14 influences alphaherpesvirus proliferation by degrading viral VP16 protein via ER stress-triggered selective autophagy. Autophagy.

[81] Schneider S.M., Lee B.H., Nicola A.V. (2021). Viral entry and the ubiquitin-proteasome system. Cell Microbiol..

[82] Luo H. (2016). Interplay between the virus and the ubiquitin-proteasome system: Molecular mechanism of viral pathogenesis. Curr. Opin. Virol..

[83] Dubiella U., Serrano I. (2021). The Ubiquitin Proteasome System as a Double Agent in Plant-Virus Interactions. Plants.

[84] Alcaide-Loridan C., Jupin I. (2012). Ubiquitin and plant viruses, let’s play together!. Plant Physiol..

[85] Gao G., Luo H. (2006). The ubiquitin-proteasome pathway in viral infections. Can. J. Physiol. Pharmacol..

[86] Isaacson M.K., Ploegh H.L. (2009). Ubiquitination, ubiquitin-like modifiers, and deubiquitination in viral infection. Cell Host Microbe.

[87] McCarthy M.K., Weinberg J.B. (2015). The immunoproteasome and viral infection: A complex regulator of inflammation. Front. Microbiol..

[88] Cetin G., Klafack S., Studencka-Turski M., Kruger E., Ebstein F. (2021). The Ubiquitin-Proteasome System in Immune Cells. Biomolecules.

[89] Bhat S.A., Vasi Z., Adhikari R., Gudur A., Ali A., Jiang L., Ferguson R., Liang D., Kuchay S. (2022). Ubiquitin proteasome system in immune regulation and therapeutics. Curr. Opin. Pharmacol..

[90] Katze M.G., He Y., Gale M. (2002). Viruses and interferon: A fight for supremacy. Nat. Rev. Immunol..

[91] Dalskov L., Gad H.H., Hartmann R. (2023). Viral recognition and the antiviral interferon response. EMBO J..

[92] Zou L., Wang X., Zhao F., Wu K., Li X., Li Z., Li Y., Chen W., Zeng S., Liu X. (2022). Viruses Hijack ERAD to Regulate Their Replication and Propagation. Int. J. Mol. Sci..

[93] Magadan J.G., Perez-Victoria F.J., Sougrat R., Ye Y., Strebel K., Bonifacino J.S. (2010). Multilayered mechanism of CD4 downregulation by HIV-1 Vpu involving distinct ER retention and ERAD targeting steps. PLoS Pathog..

[94] van den Boomen D.J., Lehner P.J. (2015). Identifying the ERAD ubiquitin E3 ligases for viral and cellular targeting of MHC class I. Mol. Immunol..

[95] Lazar C., Macovei A., Petrescu S., Branza-Nichita N. (2012). Activation of ERAD pathway by human hepatitis B virus modulates viral and subviral particle production. PLoS ONE.

[96] Park E.S., Dezhbord M., Lee A.R., Kim K.H. (2022). The Roles of Ubiquitination in Pathogenesis of Influenza Virus Infection. Int. J. Mol. Sci..

[97] Cohen F.S. (2016). How Viruses Invade Cells. Biophys. J..

[98] Herrmann C., Dybas J.M., Liddle J.C., Price A.M., Hayer K.E., Lauman R., Purman C.E., Charman M., Kim E.T., Garcia B.A. (2020). Adenovirus-mediated ubiquitination alters protein-RNA binding and aids viral RNA processing. Nat. Microbiol..

[99] Cai D., Liu L., Tian B., Fu X., Yang Q., Chen J., Zhang Y., Fang J., Shen L., Wang Y. (2022). Dual-Role Ubiquitination Regulation Shuttling the Entire Life Cycle of the Flaviviridae. Front. Microbiol..

[100] Valerdi K.M., Hage A., van Tol S., Rajsbaum R., Giraldo M.I. (2021). The Role of the Host Ubiquitin System in Promoting Replication of Emergent Viruses. Viruses.

[101] Imbert F., Langford D. (2021). Viruses, SUMO, and immunity: The interplay between viruses and the host SUMOylation system. J. Neurovirol..

[102] Zhang Q., Jia Q., Gao W., Zhang W. (2022). The Role of Deubiquitinases in Virus Replication and Host Innate Immune Response. Front. Microbiol..

[103] Bailey-Elkin B.A., Knaap R.C.M., Kikkert M., Mark B.L. (2017). Structure and Function of Viral Deubiquitinating Enzymes. J. Mol. Biol..

[104] Raaben M., Posthuma C.C., Verheije M.H., te Lintelo E.G., Kikkert M., Drijfhout J.W., Snijder E.J., Rottier P.J., de Haan C.A. (2010). The ubiquitin-proteasome system plays an important role during various stages of the coronavirus infection cycle. J. Virol..

[105] Che Y., Jiang D., Zhang Y., Zhang J., Xu T., Sun Y., Fan J., Wang J., Chang N., Wu Y. (2022). Elevated ubiquitination contributes to protective immunity against severe SARS-CoV-2 infection. Clin. Transl. Med..

[106] Maimaitiyiming Y., Yang T., Wang Q.Q., Feng Y., Chen Z., Bjorklund M., Wang F., Hu C., Hsu C.H., Naranmandura H. (2022). Heat Treatment Promotes Ubiquitin-Mediated Proteolysis of SARS-CoV-2 RNA Polymerase and Decreases Viral Load. Research.

[107] Baskol G., Ozel M., Saracoglu H., Ulger B., Kalin Unuvar G., Onuk S., Bayram A., Karayol Akin A., Muhtaroglu S., Sagiroglu P. (2022). New Avenues to Explore in SARS-CoV-2 Infection: Both TRIM25 and TRIM56 Positively Correlate with VEGF, GAS6, and sAXL in COVID-19 Patients. Viral Immunol..

[108] Zhu Y., Afolabi L.O., Wan X., Shim J.S., Chen L. (2022). TRIM family proteins: Roles in proteostasis and neurodegenerative diseases. Open Biol..

[109] Vanderboom P.M., Mun D.G., Madugundu A.K., Mangalaparthi K.K., Saraswat M., Garapati K., Chakraborty R., Ebihara H., Sun J., Pandey A. (2021). Proteomic Signature of Host Response to SARS-CoV-2 Infection in the Nasopharynx. Mol. Cell. Proteom..

[110] Xu G., Wu Y., Xiao T., Qi F., Fan L., Zhang S., Zhou J., He Y., Gao X., Zeng H. (2022). Multiomics approach reveals the ubiquitination-specific processes hijacked by SARS-CoV-2. Signal Transduct. Target. Ther..

[111] Longhitano L., Tibullo D., Giallongo C., Lazzarino G., Tartaglia N., Galimberti S., Li Volti G., Palumbo G.A., Liso A. (2020). Proteasome Inhibitors as a Possible Therapy for SARS-CoV-2. Int. J. Mol. Sci..

[112] Grosse M., Setz C., Rauch P., Auth J., Morokutti-Kurz M., Temchura V., Schubert U. (2022). Inhibitors of Deubiquitinating Enzymes Interfere with the SARS-CoV-2 Papain-like Protease and Block Virus Replication In Vitro. Viruses.

[113] Chen D., Zhao Y.G., Zhang H. (2022). Endomembrane remodeling in SARS-CoV-2 infection. Cell Insight.

[114] Clemente V., D’Arcy P., Bazzaro M. (2020). Deubiquitinating Enzymes in Coronaviruses and Possible Therapeutic Opportunities for COVID-19. Int. J. Mol. Sci..

[115] Freitas B.T., Durie I.A., Murray J., Longo J.E., Miller H.C., Crich D., Hogan R.J., Tripp R.A., Pegan S.D. (2020). Characterization and Noncovalent Inhibition of the Deubiquitinase and deISGylase Activity of SARS-CoV-2 Papain-Like Protease. ACS Infect. Dis..

[116] Narayanan A., Narwal M., Majowicz S.A., Varricchio C., Toner S.A., Ballatore C., Brancale A., Murakami K.S., Jose J. (2022). Identification of SARS-CoV-2 inhibitors targeting Mpro and PLpro using in-cell-protease assay. Commun. Biol..

[117] Mizushima N., Levine B. (2010). Autophagy in mammalian development and differentiation. Nat. Cell Biol..

[118] Parzych K.R., Klionsky D.J. (2014). An overview of autophagy: Morphology, mechanism, and regulation. Antioxid. Redox Signal..

[119] Levine B., Kroemer G. (2008). SnapShot: Macroautophagy. Cell.

[120] Li W.W., Li J., Bao J.K. (2012). Microautophagy: Lesser-known self-eating. Cell. Mol. Life Sci..

[121] Bejarano E., Cuervo A.M. (2010). Chaperone-mediated autophagy. Proc. Am. Thorac. Soc..

[122] Feng Y., He D., Yao Z., Klionsky D.J. (2014). The machinery of macroautophagy. Cell Res..

[123] Wang L., Klionsky D.J., Shen H.M. (2023). The emerging mechanisms and functions of microautophagy. Nat. Rev. Mol. Cell Biol..

[124] Kaushik S., Cuervo A.M. (2018). The coming of age of chaperone-mediated autophagy. Nat. Rev. Mol. Cell Biol..

[125] Gavilan E., Pintado C., Gavilan M.P., Daza P., Sanchez-Aguayo I., Castano A., Ruano D. (2015). Age-related dysfunctions of the autophagy lysosomal pathway in hippocampal pyramidal neurons under proteasome stress. Neurobiol. Aging.

[126] He C., Klionsky D.J. (2006). Autophagy and neurodegeneration. ACS Chem. Biol..

[127] Aman Y., Schmauck-Medina T., Hansen M., Morimoto R.I., Simon A.K., Bjedov I., Palikaras K., Simonsen A., Johansen T., Tavernarakis N. (2021). Autophagy in healthy aging and disease. Nat. Aging.

[128] Chen T., Tu S., Ding L., Jin M., Chen H., Zhou H. (2023). The role of autophagy in viral infections. J. Biomed. Sci..

[129] Mao J., Lin E., He L., Yu J., Tan P., Zhou Y. (2019). Autophagy and Viral Infection. Adv. Exp. Med. Biol..

[130] Choi Y., Bowman J.W., Jung J.U. (2018). Autophagy during viral infection—A double-edged sword. Nat. Rev. Microbiol..

[131] Rubinsztein D.C., Codogno P., Levine B. (2012). Autophagy modulation as a potential therapeutic target for diverse diseases. Nat. Rev. Drug Discov..

[132] Liang S., Wu Y.S., Li D.Y., Tang J.X., Liu H.F. (2021). Autophagy in Viral Infection and Pathogenesis. Front. Cell Dev. Biol..

[133] Orvedahl A., MacPherson S., Sumpter R., Talloczy Z., Zou Z., Levine B. (2010). Autophagy protects against Sindbis virus infection of the central nervous system. Cell Host Microbe.

[134] Wu Y., Zhou T., Hu J., Liu Y., Jin S., Wu J., Guan X., Cui J. (2022). Autophagy Activation Induces p62-Dependent Autophagic Degradation of Dengue Virus Capsid Protein During Infection. Front. Microbiol..

[135] Staring J., von Castelmur E., Blomen V.A., van den Hengel L.G., Brockmann M., Baggen J., Thibaut H.J., Nieuwenhuis J., Janssen H., van Kuppeveld F.J. (2017). PLA2G16 represents a switch between entry and clearance of Picornaviridae. Nature.

[136] Delorme-Axford E., Klionsky D.J. (2019). Inflammatory-dependent Sting activation induces antiviral autophagy to limit zika virus in the *Drosophila* brain. Autophagy.

[137] Oynebraten I. (2020). Involvement of autophagy in MHC class I antigen presentation. Scand. J. Immunol..

[138] Munz C. (2016). Autophagy Beyond Intracellular MHC Class II Antigen Presentation. Trends Immunol..

[139] Tian Y., Wang M.L., Zhao J. (2019). Crosstalk between Autophagy and Type I Interferon Responses in Innate Antiviral Immunity. Viruses.

[140] Chawla K., Subramanian G., Rahman T., Fan S., Chakravarty S., Gujja S., Demchak H., Chakravarti R., Chattopadhyay S. (2022). Autophagy in Virus Infection: A Race between Host Immune Response and Viral Antagonism. Immuno.

[141] Joubert P.E., Meiffren G., Gregoire I.P., Pontini G., Richetta C., Flacher M., Azocar O., Vidalain P.O., Vidal M., Lotteau V. (2009). Autophagy induction by the pathogen receptor CD46. Cell Host Microbe.

[142] Gregoire I.P., Richetta C., Meyniel-Schicklin L., Borel S., Pradezynski F., Diaz O., Deloire A., Azocar O., Baguet J., Le Breton M. (2011). IRGM is a common target of RNA viruses that subvert the autophagy network. PLoS Pathog..

[143] Orvedahl A., Alexander D., Talloczy Z., Sun Q., Wei Y., Zhang W., Burns D., Leib D.A., Levine B. (2007). HSV-1 ICP34.5 confers neurovirulence by targeting the Beclin 1 autophagy protein. Cell Host Microbe.

[144] Mouna L., Hernandez E., Bonte D., Brost R., Amazit L., Delgui L.R., Brune W., Geballe A.P., Beau I., Esclatine A. (2016). Analysis of the role of autophagy inhibition by two complementary human cytomegalovirus BECN1/Beclin 1-binding proteins. Autophagy.

[145] Bhatt A.P., Damania B. (2012). AKTivation of PI3K/AKT/mTOR signaling pathway by KSHV. Front. Immunol..

[146] Prentice E., Jerome W.G., Yoshimori T., Mizushima N., Denison M.R. (2004). Coronavirus replication complex formation utilizes components of cellular autophagy. J. Biol. Chem..

[147] Paul D., Madan V., Ramirez O., Bencun M., Stoeck I.K., Jirasko V., Bartenschlager R. (2018). Glycine Zipper Motifs in Hepatitis C Virus Nonstructural Protein 4B Are Required for the Establishment of Viral Replication Organelles. J. Virol..

[148] Gannage M., Dormann D., Albrecht R., Dengjel J., Torossi T., Ramer P.C., Lee M., Strowig T., Arrey F., Conenello G. (2009). Matrix protein 2 of influenza A virus blocks autophagosome fusion with lysosomes. Cell Host Microbe.

[149] Ding B., Zhang G., Yang X., Zhang S., Chen L., Yan Q., Xu M., Banerjee A.K., Chen M. (2014). Phosphoprotein of human parainfluenza virus type 3 blocks autophagosome-lysosome fusion to increase virus production. Cell Host Microbe.

[150] Wu Y.W., Mettling C., Wu S.R., Yu C.Y., Perng G.C., Lin Y.S., Lin Y.L. (2016). Autophagy-associated dengue vesicles promote viral transmission avoiding antibody neutralization. Sci. Rep..

[151] Chen Y.H., Du W., Hagemeijer M.C., Takvorian P.M., Pau C., Cali A., Brantner C.A., Stempinski E.S., Connelly P.S., Ma H.C. (2015). Phosphatidylserine vesicles enable efficient en bloc transmission of enteroviruses. Cell.

[152] Jin S., He X., Ma L., Zhuang Z., Wang Y., Lin M., Cai S., Wei L., Wang Z., Zhao Z. (2022). Suppression of ACE2 SUMOylation protects against SARS-CoV-2 infection through TOLLIP-mediated selective autophagy. Nat. Commun..

[153] Li F., Li J., Wang P.H., Yang N., Huang J., Ou J., Xu T., Zhao X., Liu T., Huang X. (2021). SARS-CoV-2 spike promotes inflammation and apoptosis through autophagy by ROS-suppressed PI3K/AKT/mTOR signaling. Biochim. Biophys. Acta Mol. Basis Dis..

[154] Silva R., Travassos L.H. (2022). Autophagy: A self-guard against SARS-CoV-2. Med. Hypotheses.

[155] Gassen N.C., Papies J., Bajaj T., Emanuel J., Dethloff F., Chua R.L., Trimpert J., Heinemann N., Niemeyer C., Weege F. (2021). SARS-CoV-2-mediated dysregulation of metabolism and autophagy uncovers host-targeting antivirals. Nat. Commun..

[156] Rayner J.O., Roberts R.A., Kim J., Poklepovic A., Roberts J.L., Booth L., Dent P. (2020). AR12 (OSU-03012) suppresses GRP78 expression and inhibits SARS-CoV-2 replication. Biochem. Pharmacol..

[157] Li M., Ferretti M., Ying B., Descamps H., Lee E., Dittmar M., Lee J.S., Whig K., Kamalia B., Dohnalova L. (2021). Pharmacological activation of STING blocks SARS-CoV-2 infection. Sci. Immunol..

[158] Tomic S., Dokic J., Stevanovic D., Ilic N., Gruden-Movsesijan A., Dinic M., Radojevic D., Bekic M., Mitrovic N., Tomasevic R. (2021). Reduced Expression of Autophagy Markers and Expansion of Myeloid-Derived Suppressor Cells Correlate with Poor T Cell Response in Severe COVID-19 Patients. Front. Immunol..

[159] Shang C., Zhuang X., Zhang H., Li Y., Zhu Y., Lu J., Ge C., Cong J., Li T., Li N. (2021). Inhibition of Autophagy Suppresses SARS-CoV-2 Replication and Ameliorates Pneumonia in hACE2 Transgenic Mice and Xenografted Human Lung Tissues. J. Virol..

[160] Verma R., Saha S., Kumar S., Mani S., Maiti T.K., Surjit M. (2021). RNA-Protein Interaction Analysis of SARS-CoV-2 5’ and 3’ Untranslated Regions Reveals a Role of Lysosome-Associated Membrane Protein-2a during Viral Infection. mSystems.

[161] Zhou H., Hu Z., Castro-Gonzalez S. (2023). Bidirectional interplay between SARS-CoV-2 and autophagy. mBio.

[162] Ivanova T., Mariienko Y., Mehterov N., Kazakova M., Sbirkov Y., Todorova K., Hayrabedyan S., Sarafian V. (2023). Autophagy and SARS-CoV-2-Old Players in New Games. Int. J. Mol. Sci..

[163] Tan X., Cai K., Li J., Yuan Z., Chen R., Xiao H., Xu C., Hu B., Qin Y., Ding B. (2023). Coronavirus subverts ER-phagy by hijacking FAM134B and ATL3 into p62 condensates to facilitate viral replication. Cell Rep..

[164] Samimi N., Farjam M., Klionsky D.J., Rezaei N. (2022). The role of autophagy in the pathogenesis of SARS-CoV-2 infection in different cell types. Autophagy.

[165] Zhang Y., Sun H., Pei R., Mao B., Zhao Z., Li H., Lin Y., Lu K. (2021). The SARS-CoV-2 protein ORF3a inhibits fusion of autophagosomes with lysosomes. Cell Discov..

[166] Zhang Y., Chen Y., Li Y., Huang F., Luo B., Yuan Y., Xia B., Ma X., Yang T., Yu F. (2021). The ORF8 protein of SARS-CoV-2 mediates immune evasion through down-regulating MHC-Iota. Proc. Natl. Acad. Sci. USA.

[167] Sargazi S., Sheervalilou R., Rokni M., Shirvaliloo M., Shahraki O., Rezaei N. (2021). The role of autophagy in controlling SARS-CoV-2 infection: An overview on virophagy-mediated molecular drug targets. Cell Biol. Int..

[168] Hayn M., Hirschenberger M., Koepke L., Nchioua R., Straub J.H., Klute S., Hunszinger V., Zech F., Prelli Bozzo C., Aftab W. (2021). Systematic functional analysis of SARS-CoV-2 proteins uncovers viral innate immune antagonists and remaining vulnerabilities. Cell Rep..

[169] Zou D., Xu J., Duan X., Xu X., Li P., Cheng L., Zheng L., Li X., Zhang Y., Wang X. (2019). Porcine epidemic diarrhea virus ORF3 protein causes endoplasmic reticulum stress to facilitate autophagy. Vet. Microbiol..

[170] Mohamud Y., Xue Y.C., Liu H., Ng C.S., Bahreyni A., Jan E., Luo H. (2021). The papain-like protease of coronaviruses cleaves ULK1 to disrupt host autophagy. Biochem. Biophys. Res. Commun..

[171] Benvenuto D., Angeletti S., Giovanetti M., Bianchi M., Pascarella S., Cauda R., Ciccozzi M., Cassone A. (2020). Evolutionary analysis of SARS-CoV-2: How mutation of Non-Structural Protein 6 (NSP6) could affect viral autophagy. J. Infect..

[172] Yang N., Shen H.M. (2020). Targeting the Endocytic Pathway and Autophagy Process as a Novel Therapeutic Strategy in COVID-19. Int. J. Biol. Sci..

[173] Liang H., Luo D., Liao H., Li S. (2022). Coronavirus Usurps the Autophagy-Lysosome Pathway and Induces Membranes Rearrangement for Infection and Pathogenesis. Front. Microbiol..

[174] Qu Y., Wang X., Zhu Y., Wang W., Wang Y., Hu G., Liu C., Li J., Ren S., Xiao M.Z.X. (2021). ORF3a-Mediated Incomplete Autophagy Facilitates Severe Acute Respiratory Syndrome Coronavirus-2 Replication. Front. Cell Dev. Biol..

[175] Koepke L., Hirschenberger M., Hayn M., Kirchhoff F., Sparrer K.M. (2021). Manipulation of autophagy by SARS-CoV-2 proteins. Autophagy.

[176] Wu W., Luo X., Ren M. (2021). Clearance or Hijack: Universal Interplay Mechanisms Between Viruses and Host Autophagy from Plants to Animals. Front. Cell. Infect. Microbiol..

[177] Hou P., Wang X., Wang H., Wang T., Yu Z., Xu C., Zhao Y., Wang W., Zhao Y., Chu F. (2023). The ORF7a protein of SARS-CoV-2 initiates autophagy and limits autophagosome-lysosome fusion via degradation of SNAP29 to promote virus replication. Autophagy.

[178] Li X., Hou P., Ma W., Wang X., Wang H., Yu Z., Chang H., Wang T., Jin S., Wang X. (2022). SARS-CoV-2 ORF10 suppresses the antiviral innate immune response by degrading MAVS through mitophagy. Cell. Mol. Immunol..

[179] Ouyang L., Gong J. (2020). Mitochondrial-targeted ubiquinone: A potential treatment for COVID-19. Med. Hypotheses.

[180] Domizio J.D., Gulen M.F., Saidoune F., Thacker V.V., Yatim A., Sharma K., Nass T., Guenova E., Schaller M., Conrad C. (2022). The cGAS-STING pathway drives type I IFN immunopathology in COVID-19. Nature.

[181] Su J., Shen S., Hu Y., Chen S., Cheng L., Cai Y., Wei W., Wang Y., Rui Y., Yu X.F. (2023). SARS-CoV-2 ORF3a inhibits cGAS-STING-mediated autophagy flux and antiviral function. J. Med. Virol..

[182] Han L., Zheng Y., Deng J., Nan M.L., Xiao Y., Zhuang M.W., Zhang J., Wang W., Gao C., Wang P.H. (2022). SARS-CoV-2 ORF10 antagonizes STING-dependent interferon activation and autophagy. J. Med. Virol..

[183] Madiraju C., Novack J.P., Reed J.C., Matsuzawa S.I. (2022). K63 ubiquitination in immune signaling. Trends Immunol..

[184] Rui Y., Su J., Shen S., Hu Y., Huang D., Zheng W., Lou M., Shi Y., Wang M., Chen S. (2021). Unique and complementary suppression of cGAS-STING and RNA sensing- triggered innate immune responses by SARS-CoV-2 proteins. Signal Transduct. Target. Ther..

[185] Hartl F.U., Bracher A., Hayer-Hartl M. (2011). Molecular chaperones in protein folding and proteostasis. Nature.

[186] Adriaenssens E., Asselbergh B., Rivera-Mejias P., Bervoets S., Vendredy L., De Winter V., Spaas K., de Rycke R., van Isterdael G., Impens F. (2023). Small heat shock proteins operate as molecular chaperones in the mitochondrial intermembrane space. Nat. Cell Biol..

[187] Bobori C., Theocharopoulou G., Vlamos P. (2017). Molecular Chaperones in Neurodegenerative Diseases: A Short Review. Adv. Exp. Med. Biol..

[188] Morimoto R.I. (2008). Proteotoxic stress and inducible chaperone networks in neurodegenerative disease and aging. Genes. Dev..

[189] Witt S.N. (2013). Molecular chaperones, alpha-synuclein, and neurodegeneration. Mol. Neurobiol..

[190] Franklin T.B., Krueger-Naug A.M., Clarke D.B., Arrigo A.P., Currie R.W. (2005). The role of heat shock proteins Hsp70 and Hsp27 in cellular protection of the central nervous system. Int. J. Hyperth..

[191] Bohush A., Bieganowski P., Filipek A. (2019). Hsp90 and Its Co-Chaperones in Neurodegenerative Diseases. Int. J. Mol. Sci..

[192] Hooper P.L., Hightower L.E., Hooper P.L. (2012). Loss of stress response as a consequence of viral infection: Implications for disease and therapy. Cell Stress Chaperones.

[193] Paladino L., Vitale A.M., Caruso Bavisotto C., Conway de Macario E., Cappello F., Macario A.J.L., Gammazza A.M. (2020). The Role of Molecular Chaperones in Virus Infection and Implications for Understanding and Treating COVID-19. J. Clin. Med..

[194] Cheng X., Belshan M., Ratner L. (2008). Hsp40 facilitates nuclear import of the human immunodeficiency virus type 2 Vpx-mediated preintegration complex. J. Virol..

[195] Oglesbee M.J., Pratt M., Carsillo T. (2002). Role for heat shock proteins in the immune response to measles virus infection. Viral Immunol..

[196] Tanaka Y., Kanai F., Kawakami T., Tateishi K., Ijichi H., Kawabe T., Arakawa Y., Kawakami T., Nishimura T., Shirakata Y. (2004). Interaction of the hepatitis B virus X protein (HBx) with heat shock protein 60 enhances HBx-mediated apoptosis. Biochem. Biophys. Res. Commun..

[197] Kang S.M., Kim S.J., Kim J.H., Lee W., Kim G.W., Lee K.H., Choi K.Y., Oh J.W. (2009). Interaction of hepatitis C virus core protein with Hsp60 triggers the production of reactive oxygen species and enhances TNF-α-mediated apoptosis. Cancer Lett..

[198] Zhang X., Yu W. (2022). Heat shock proteins and viral infection. Front. Immunol..

[199] Wang Z., Li Y., Yang X., Zhao J., Cheng Y., Wang J. (2020). Mechanism and Complex Roles of HSC70 in Viral Infections. Front. Microbiol..

[200] Hauser H., Shen L., Gu Q.L., Krueger S., Chen S.Y. (2004). Secretory heat-shock protein as a dendritic cell-targeting molecule: A new strategy to enhance the potency of genetic vaccines. Gene Ther..

[201] Takashima K., Oshiumi H., Matsumoto M., Seya T. (2018). DNAJB1/HSP40 Suppresses Melanoma Differentiation-Associated Gene 5-Mitochondrial Antiviral Signaling Protein Function in Conjunction with HSP70. J. Innate Immun..

[202] Sun M., Yu Z., Ma J., Pan Z., Lu C., Yao H. (2017). Down-regulating heat shock protein 27 is involved in porcine epidemic diarrhea virus escaping from host antiviral mechanism. Vet. Microbiol..

[203] Le Y., Jia P., Jin Y., Liu W., Jia K., Yi M. (2017). The antiviral role of heat shock protein 27 against red spotted grouper nervous necrosis virus infection in sea perch. Fish. Shellfish. Immunol..

[204] Kumar M., Rawat P., Khan S.Z., Dhamija N., Chaudhary P., Ravi D.S., Mitra D. (2011). Reciprocal regulation of human immunodeficiency virus-1 gene expression and replication by heat shock proteins 40 and 70. J. Mol. Biol..

[205] Batra J., Tripathi S., Kumar A., Katz J.M., Cox N.J., Lal R.B., Sambhara S., Lal S.K. (2016). Human Heat shock protein 40 (Hsp40/DnaJB1) promotes influenza A virus replication by assisting nuclear import of viral ribonucleoproteins. Sci. Rep..

[206] Park S.G., Lee S.M., Jung G. (2003). Antisense oligodeoxynucleotides targeted against molecular chaperonin Hsp60 block human hepatitis B virus replication. J. Biol. Chem..

[207] Wan Q., Song D., Li H., He M.L. (2020). Stress proteins: The biological functions in virus infection, present and challenges for target-based antiviral drug development. Signal Transduct. Target. Ther..

[208] Reyes-Del Valle J., Chavez-Salinas S., Medina F., Del Angel R.M. (2005). Heat shock protein 90 and heat shock protein 70 are components of dengue virus receptor complex in human cells. J. Virol..

[209] Thongtan T., Wikan N., Wintachai P., Rattanarungsan C., Srisomsap C., Cheepsunthorn P., Smith D.R. (2012). Characterization of putative Japanese encephalitis virus receptor molecules on microglial cells. J. Med. Virol..

[210] Lubkowska A., Pluta W., Stronska A., Lalko A. (2021). Role of Heat Shock Proteins (HSP70 and HSP90) in Viral Infection. Int. J. Mol. Sci..

[211] Okamoto T., Nishimura Y., Ichimura T., Suzuki K., Miyamura T., Suzuki T., Moriishi K., Matsuura Y. (2006). Hepatitis C virus RNA replication is regulated by FKBP8 and Hsp90. EMBO J..

[212] Chase G., Deng T., Fodor E., Leung B.W., Mayer D., Schwemmle M., Brownlee G. (2008). Hsp90 inhibitors reduce influenza virus replication in cell culture. Virology.

[213] Aviner R., Frydman J. (2020). Proteostasis in Viral Infection: Unfolding the Complex Virus-Chaperone Interplay. Cold Spring Harb. Perspect. Biol..

[214] Kumar M., Mitra D. (2005). Heat shock protein 40 is necessary for human immunodeficiency virus-1 Nef-mediated enhancement of viral gene expression and replication. J. Biol. Chem..

[215] Shelton M.N., Huang M.B., Ali S.A., Powell M.D., Bond V.C. (2012). Secretion modification region-derived peptide disrupts HIV-1 Nef’s interaction with mortalin and blocks virus and Nef exosome release. J. Virol..

[216] Geiger R., Andritschke D., Friebe S., Herzog F., Luisoni S., Heger T., Helenius A. (2011). BAP31 and BiP are essential for dislocation of SV40 from the endoplasmic reticulum to the cytosol. Nat. Cell Biol..

[217] Khongwichit S., Sornjai W., Jitobaom K., Greenwood M., Greenwood M.P., Hitakarun A., Wikan N., Murphy D., Smith D.R. (2021). A functional interaction between GRP78 and Zika virus E protein. Sci. Rep..

[218] Chu H., Chan C.M., Zhang X., Wang Y., Yuan S., Zhou J., Au-Yeung R.K., Sze K.H., Yang D., Shuai H. (2018). Middle East respiratory syndrome coronavirus and bat coronavirus HKU9 both can utilize GRP78 for attachment onto host cells. J. Biol. Chem..

[219] Elfiky A.A., Amr A., Mosaad A., Mubarak A.K., Sayed M.A., El-Halwany K.K. (2023). Cs-GRP78 recognition site on dengue virus envelope protein: In silico perspective. Future Virol..

[220] Buchkovich N.J., Maguire T.G., Yu Y., Paton A.W., Paton J.C., Alwine J.C. (2008). Human cytomegalovirus specifically controls the levels of the endoplasmic reticulum chaperone BiP/GRP78, which is required for virion assembly. J. Virol..

[221] Maruri-Avidal L., Lopez S., Arias C.F. (2008). Endoplasmic reticulum chaperones are involved in the morphogenesis of rotavirus infectious particles. J. Virol..

[222] Fislova T., Thomas B., Graef K.M., Fodor E. (2010). Association of the influenza virus RNA polymerase subunit PB2 with the host chaperonin CCT. J. Virol..

[223] Zhang J., Ye C., Ruan X., Zan J., Xu Y., Liao M., Zhou J. (2014). The chaperonin CCTα is required for efficient transcription and replication of rabies virus. Microbiol. Immunol..

[224] Knowlton J.J., Fernandez de Castro I., Ashbrook A.W., Gestaut D.R., Zamora P.F., Bauer J.A., Forrest J.C., Frydman J., Risco C., Dermody T.S. (2018). The TRiC chaperonin controls reovirus replication through outer-capsid folding. Nat. Microbiol..

[225] Tahmaz I., Shahmoradi Ghahe S., Topf U. (2022). Prefoldin Function in Cellular Protein Homeostasis and Human Diseases. Front. Cell Dev. Biol..

[226] Knowlton J.J., Gestaut D., Ma B., Taylor G., Seven A.B., Leitner A., Wilson G.J., Shanker S., Yates N.A., Prasad B.V.V. (2021). Structural and functional dissection of reovirus capsid folding and assembly by the prefoldin-TRiC/CCT chaperone network. Proc. Natl. Acad. Sci. USA.

[227] Tsao M.L., Chao C.H., Yeh C.T. (2006). Interaction of hepatitis C virus F protein with prefoldin 2 perturbs tubulin cytoskeleton organization. Biochem. Biophys. Res. Commun..

[228] Rain J.C., Cribier A., Gerard A., Emiliani S., Benarous R. (2009). Yeast two-hybrid detection of integrase-host factor interactions. Methods.

[229] Li C., Chu H., Liu X., Chiu M.C., Zhao X., Wang D., Wei Y., Hou Y., Shuai H., Cai J. (2020). Human coronavirus dependency on host heat shock protein 90 reveals an antiviral target. Emerg. Microbes Infect..

[230] Zhao Z., Xu L.D., Zhang F., Liang Q.Z., Jiao Y., Shi F.S., He B., Xu P., Huang Y.W. (2023). Heat shock protein 90 facilitates SARS-CoV-2 structural protein-mediated virion assembly and promotes virus-induced pyroptosis. J. Biol. Chem..

[231] Wyler E., Mosbauer K., Franke V., Diag A., Gottula L.T., Arsie R., Klironomos F., Koppstein D., Honzke K., Ayoub S. (2021). Transcriptomic profiling of SARS-CoV-2 infected human cell lines identifies HSP90 as target for COVID-19 therapy. iScience.

[232] Sabirli R., Koseler A., Goren T., Turkcuer I., Kurt O. (2021). High GRP78 levels in COVID-19 infection: A case-control study. Life Sci..

[233] Shin J., Toyoda S., Fukuhara A., Shimomura I. (2022). GRP78, a Novel Host Factor for SARS-CoV-2: The Emerging Roles in COVID-19 Related to Metabolic Risk Factors. Biomedicines.

[234] Carlos A.J., Ha D.P., Yeh D.W., Van Krieken R., Tseng C.C., Zhang P., Gill P., Machida K., Lee A.S. (2021). The chaperone GRP78 is a host auxiliary factor for SARS-CoV-2 and GRP78 depleting antibody blocks viral entry and infection. J. Biol. Chem..

[235] Shin W.J., Ha D.P., Machida K., Lee A.S. (2022). The stress-inducible ER chaperone GRP78/BiP is upregulated during SARS-CoV-2 infection and acts as a pro-viral protein. Nat. Commun..

[236] Ha D.P., Shin W.J., Hernandez J.C., Neamati N., Dubeau L., Machida K., Lee A.S. (2023). GRP78 Inhibitor YUM70 Suppresses SARS-CoV-2 Viral Entry, Spike Protein Production and Ameliorates Lung Damage. Viruses.

[237] Zhang L., Zhou L., Bao L., Liu J., Zhu H., Lv Q., Liu R., Chen W., Tong W., Wei Q. (2021). SARS-CoV-2 crosses the blood-brain barrier accompanied with basement membrane disruption without tight junctions alteration. Signal Transduct. Target. Ther..

[238] Butowt R., Meunier N., Bryche B., von Bartheld C.S. (2021). The olfactory nerve is not a likely route to brain infection in COVID-19: A critical review of data from humans and animal models. Acta Neuropathol..

[239] Villadiego J., Garcia-Arriaza J., Ramirez-Lorca R., Garcia-Swinburn R., Cabello-Rivera D., Rosales-Nieves A.E., Alvarez-Vergara M.I., Cala-Fernandez F., Garcia-Roldan E., Lopez-Ogayar J.L. (2023). Full protection from SARS-CoV-2 brain infection and damage in susceptible transgenic mice conferred by MVA-CoV2-S vaccine candidate. Nat. Neurosci..

[240] Tavassoly O., Safavi F., Tavassoly I. (2020). Seeding Brain Protein Aggregation by SARS-CoV-2 as a Possible Long-Term Complication of COVID-19 Infection. ACS Chem. Neurosci..

[241] Butowt R., von Bartheld C.S. (2022). The route of SARS-CoV-2 to brain infection: Have we been barking up the wrong tree?. Mol. Neurodegener..

[242] Sollmann N., Beer A.J., Kirchhoff F. (2022). SARS-CoV-2 infection and the brain: Direct evidence for brain changes in milder cases. Signal Transduct. Target. Ther..

[243] Jung J., Michalak M., Agellon L.B. (2017). Endoplasmic Reticulum Malfunction in the Nervous System. Front. Neurosci..

[244] Choi I., Zhang Y., Seegobin S.P., Pruvost M., Wang Q., Purtell K., Zhang B., Yue Z. (2020). Microglia clear neuron-released α-synuclein via selective autophagy and prevent neurodegeneration. Nat. Commun..

[245] Srimat Kandadai K., Kotur M.B., Dokalis N., Amrein I., Keller C.W., Munz C., Wolfer D., Prinz M., Lunemann J.D. (2021). ATG5 in microglia does not contribute vitally to autoimmune neuroinflammation in mice. Autophagy.

[246] Tydlacka S., Wang C.E., Wang X., Li S., Li X.J. (2008). Differential activities of the ubiquitin-proteasome system in neurons versus glia may account for the preferential accumulation of misfolded proteins in neurons. J. Neurosci..

[247] Subramaniam S. (2019). Selective Neuronal Death in Neurodegenerative Diseases: The Ongoing Mystery. Yale J. Biol. Med..

[248] Bedford L., Hay D., Devoy A., Paine S., Powe D.G., Seth R., Gray T., Topham I., Fone K., Rezvani N. (2008). Depletion of 26S proteasomes in mouse brain neurons causes neurodegeneration and Lewy-like inclusions resembling human pale bodies. J. Neurosci..

[249] Komatsu M., Kominami E., Tanaka K. (2006). Autophagy and neurodegeneration. Autophagy.

[250] Hara T., Nakamura K., Matsui M., Yamamoto A., Nakahara Y., Suzuki-Migishima R., Yokoyama M., Mishima K., Saito I., Okano H. (2006). Suppression of basal autophagy in neural cells causes neurodegenerative disease in mice. Nature.

[251] Bjornevik K., Cortese M., Healy B.C., Kuhle J., Mina M.J., Leng Y., Elledge S.J., Niebuhr D.W., Scher A.I., Munger K.L. (2022). Longitudinal analysis reveals high prevalence of Epstein-Barr virus associated with multiple sclerosis. Science.

[252] Fulop T., Witkowski J.M., Larbi A., Khalil A., Herbein G., Frost E.H. (2019). Does HIV infection contribute to increased beta-amyloid synthesis and plaque formation leading to neurodegeneration and Alzheimer’s disease?. J. Neurovirol.

[253] De Chiara G., Marcocci M.E., Sgarbanti R., Civitelli L., Ripoli C., Piacentini R., Garaci E., Grassi C., Palamara A.T. (2012). Infectious agents and neurodegeneration. Mol. Neurobiol..

[254] Kristen H., Santana S., Sastre I., Recuero M., Bullido M.J., Aldudo J. (2015). Herpes simplex virus type 2 infection induces AD-like neurodegeneration markers in human neuroblastoma cells. Neurobiol. Aging.

[255] Bortolotti D., Gentili V., Rotola A., Caselli E., Rizzo R. (2019). HHV-6A infection induces amyloid-beta expression and activation of microglial cells. Alzheimers Res. Ther..

[256] Levine K.S., Leonard H.L., Blauwendraat C., Iwaki H., Johnson N., Bandres-Ciga S., Ferrucci L., Faghri F., Singleton A.B., Nalls M.A. (2023). Virus exposure and neurodegenerative disease risk across national biobanks. Neuron.

[257] Leblanc P., Vorberg I.M. (2022). Viruses in neurodegenerative diseases: More than just suspects in crimes. PLoS Pathog..

[258] Romeo M.A., Gilardini Montani M.S., Gaeta A., D’Orazi G., Faggioni A., Cirone M. (2020). HHV-6A infection dysregulates autophagy/UPR interplay increasing beta amyloid production and tau phosphorylation in astrocytoma cells as well as in primary neurons, possible molecular mechanisms linking viral infection to Alzheimer’s disease. Biochim. Biophys. Acta Mol. Basis Dis..

[259] Marreiros R., Muller-Schiffmann A., Trossbach S.V., Prikulis I., Hansch S., Weidtkamp-Peters S., Moreira A.R., Sahu S., Soloviev I., Selvarajah S. (2020). Disruption of cellular proteostasis by H1N1 influenza A virus causes α-synuclein aggregation. Proc. Natl. Acad. Sci. USA.

[260] Rieder A.S., Deniz B.F., Netto C.A., Wyse A.T.S. (2022). A Review of In Silico Research, SARS-CoV-2, and Neurodegeneration: Focus on Papain-Like Protease. Neurotox. Res..

[261] Karvandi M.S., Sheikhzadeh Hesari F., Aref A.R., Mahdavi M. (2023). The neuroprotective effects of targeting key factors of neuronal cell death in neurodegenerative diseases: The role of ER stress, oxidative stress, and neuroinflammation. Front. Cell. Neurosci..

[262] Rifino N., Censori B., Agazzi E., Alimonti D., Bonito V., Camera G., Conti M.Z., Foresti C., Frigeni B., Gerevini S. (2021). Neurologic manifestations in 1760 COVID-19 patients admitted to Papa Giovanni XXIII Hospital, Bergamo, Italy. J Neurol.

[263] Natoli S., Oliveira V., Calabresi P., Maia L.F., Pisani A. (2020). Does SARS-CoV-2 invade the brain? Translational lessons from animal models. Eur. J. Neurol..

[264] Baazaoui N., Iqbal K. (2022). COVID-19 and Neurodegenerative Diseases: Prion-Like Spread and Long-Term Consequences. J. Alzheimers Dis..

[265] Krey L., Huber M.K., Hoglinger G.U., Wegner F. (2021). Can SARS-CoV-2 Infection Lead to Neurodegeneration and Parkinson’s Disease?. Brain Sci..

[266] Idrees D., Kumar V. (2021). SARS-CoV-2 spike protein interactions with amyloidogenic proteins: Potential clues to neurodegeneration. Biochem. Biophys. Res. Commun..

[267] Bowen D.R., Pathak S., Nadar R.M., Parise R.D., Ramesh S., Govindarajulu M., Moore A., Ren J., Moore T., Dhanasekaran M. (2023). Oxidative stress and COVID-19-associated neuronal dysfunction: Mechanisms and therapeutic implications. Acta Biochim. Biophys. Sin..

[268] Lingor P., Demleitner A.F., Wolff A.W., Feneberg E. (2022). SARS-CoV-2 and neurodegenerative diseases: What we know and what we don’t. J. Neural Transm..

[269] Tonges L., Klebe S. (2022). SARS-CoV-2, COVID-19 and Neurodegeneration. Brain Sci..

[270] Strong M.J. (2023). SARS-CoV-2, aging, and Post-COVID-19 neurodegeneration. J. Neurochem..

[271] Salari M., Etemadifar M. (2021). Can COVID-19 accelerate neurodegeneration?. Clin. Case Rep..

[272] Ramani A., Muller L., Ostermann P.N., Gabriel E., Abida-Islam P., Muller-Schiffmann A., Mariappan A., Goureau O., Gruell H., Walker A. (2020). SARS-CoV-2 targets neurons of 3D human brain organoids. EMBO J..

[273] Prasad K., AlOmar S.Y., Alqahtani S.A.M., Malik M.Z., Kumar V. (2021). Brain Disease Network Analysis to Elucidate the Neurological Manifestations of COVID-19. Mol. Neurobiol..

[274] Paniz-Mondolfi A., Bryce C., Grimes Z., Gordon R.E., Reidy J., Lednicky J., Sordillo E.M., Fowkes M. (2020). Central nervous system involvement by severe acute respiratory syndrome coronavirus-2 (SARS-CoV-2). J. Med. Virol..

[275] Jin M.M., Wang F., Qi D., Liu W.W., Gu C., Mao C.J., Yang Y.P., Zhao Z., Hu L.F., Liu C.F. (2018). A Critical Role of Autophagy in Regulating Microglia Polarization in Neurodegeneration. Front. Aging Neurosci..

[276] Oslowski C.M., Urano F. (2011). Measuring ER stress and the unfolded protein response using mammalian tissue culture system. Methods Enzymol..

[277] Macauslane K.L., Pegg C.L., Short K.R., Schulz B.L. (2023). Modulation of endoplasmic reticulum stress response pathways by respiratory viruses. Crit. Rev. Microbiol..

[278] Thakur A., Sharma V., Averbek S., Liang L., Pandya N., Kumar G., Cili A., Zhang K. (2023). Immune landscape and redox imbalance during neurological disorders in COVID-19. Cell Death Dis..

[279] Saleh J., Peyssonnaux C., Singh K.K., Edeas M. (2020). Mitochondria and microbiota dysfunction in COVID-19 pathogenesis. Mitochondrion.

[280] Matias-Perez D., Hernandez-Bautista E., Antonio Garcia-Montalvo I. (2022). Oxidative Stress Derived from COVID-19 and Its Possible Association with the Development of Neurodegenerative Diseases. Arch. Neurosci..

[281] Hohn A., Tramutola A., Cascella R. (2020). Proteostasis Failure in Neurodegenerative Diseases: Focus on Oxidative Stress. Oxid. Med. Cell. Longev..

[282] Holubiec M.I., Gellert M., Hanschmann E.M. (2022). Redox signaling and metabolism in Alzheimer’s disease. Front. Aging Neurosci..

[283] Passos F.R.S., Heimfarth L., Monteiro B.S., Correa C.B., Moura T.R., Araujo A.A.S., Martins-Filho P.R., Quintans-Junior L.J., Quintans J.S.S. (2022). Oxidative stress and inflammatory markers in patients with COVID-19: Potential role of RAGE, HMGB1, GFAP and COX-2 in disease severity. Int. Immunopharmacol..

